# Merits, features, and desiderata to be considered when developing electronic health records with embedded clinical decision support systems in Palestinian hospitals: a consensus study

**DOI:** 10.1186/s12911-019-0928-3

**Published:** 2019-11-08

**Authors:** Ramzi Shawahna

**Affiliations:** 1 Department of Physiology, Pharmacology and Toxicology, Faculty of Medicine and Health Sciences, An-Najah National University, Nablus, Palestine; 2 An-Najah BioSciences Unit, Centre for Poisons Control, Chemical and Biological Analyses, An-Najah National University, Nablus, Palestine

**Keywords:** Analytic hierarchy process, Clinical decision support systems, Delphi technique, Electronic health records, Health informatics, Hospitals, Medication errors, Patient safety

## Abstract

**Background:**

Electronic health records (EHRs) with embedded clinical decision support systems (CDSSs) have the potential to improve healthcare delivery. This study was conducted to explore merits, features, and desiderata to be considered when planning for, designing, developing, implementing, piloting, evaluating, maintaining, upgrading, and/or using EHRs with CDSSs.

**Methods:**

A mixed-method combining the Delphi technique and Analytic Hierarchy Process was used. Potentially important items were collected after a thorough search of the literature and from interviews with key contact experts (*n = 19*). Opinions and views of the 76 panelists on the use of EHRs were also explored. Iterative Delphi rounds were conducted to achieve consensus on 122 potentially important items by a panel of 76 participants. Items on which consensus was achieved were ranked in the order of their importance using the Analytic Hierarchy Process.

**Results:**

Of the 122 potentially important items presented to the panelists in the Delphi rounds, consensus was achieved on 110 (90.2%) items. Of these, 16 (14.5%) items were related to the demographic characteristics of the patient, 16 (14.5%) were related to prescribing medications, 16 (14.5%) were related to checking prescriptions and alerts, 14 (12.7%) items were related to the patient’s identity, 13 (11.8%) items were related to patient assessment, 12 (10.9%) items were related to the quality of alerts, 11 (10%) items were related to admission and discharge of the patient, 9 (8.2%) items were general features, and 3 (2.7%) items were related to diseases and making diagnosis.

**Conclusions:**

In this study, merits, features, and desiderata to be considered when planning for, designing, developing, implementing, piloting, evaluating, maintaining, upgrading, and/or using EHRs with CDSSs were explored. Considering items on which consensus was achieved might promote congruence and safe use of EHRs. Further studies are still needed to determine if these recommendations can improve patient safety and outcomes in Palestinian hospitals.

## Background

Improving patient safety has become a top priority in healthcare systems around the globe [1]. Recent digital innovations and advancements in information technology (IT) have radically transformed many aspects of our world today, including healthcare delivery. Electronic health records (EHRs), also known as computerized patient records have largely replaced traditional paper-based health records in many hospitals and other healthcare delivery centers around the world [2]. These EHRs allow systematic collection and compilation of patient information such as medical history, vital signs, laboratory results, medical imaging reports, prescription and non-prescription medications, and other notes made by the healthcare providers (physicians, hospital pharmacists, and nurses) [2, 3]. Many of these EHRs include clinical decision support systems (CDSSs) that can offer medical and clinical information, warnings, alerts, reminders, and/or other support services that might help the healthcare providers make decisions and improve healthcare delivery [4]. Previous studies have reported that EHRs reduced documentation times, increased adherence of the healthcare providers to guidelines, improved communication between the healthcare providers, reduced medication errors, reduced adverse drug events, improved healthcare delivery, and promoted safer healthcare practices [5–7]. Currently, there is consensus that EHRs have the potential to improve healthcare delivery and therefore their full adoption and implementation have been recommended and incentivized [6, 8].

Despite the well-established benefits of adopting and successfully implementing well-designed EHR and CDSS systems, the literature has also reported many cases in which healthcare providers were dissatisfied with many features of EHR and CDSS systems [9–11]. It has been argued that the healthcare providers spend more time interacting with computers compared to time spent interacting with their patients face-to-face [12]. This might result in compromising the quality and safety of healthcare delivery, worsening patient-healthcare provider relationship, and fatigue of healthcare providers [9].

Additionally, poor design of EHRs and CDSS systems can adversely affect patient safety and consequently might compromise the patient health outcomes [13–16]. In many cases, system failures have led to incorrect identification of patients, dose calculation errors, and inability to access essential patient clinical data [13]. Previous studies have shown that EHRs and vendors were reported as sources of harm to patients [13, 14]. Howe et al. analyzed reports voluntarily entered by nurses into the Pennsylvania Patient Safety Authority database in the period from 2013 through 2016 [14]. The study reported that 557 safety events related to usability of EHRs resulted in patient harm. Of those, 468 required monitoring to prevent harm, 80 caused temporary harm, 7 cause permanent harm, and 2 allegedly resulted in death of patients.

EHR and CDSS systems differ significantly in terms of the informational items that the healthcare providers need to collect and enter as well as the support services that such systems can provide. Khalifa reported 10 success strategies and requirements for CDSSs [17]. Roshanov et al. reported features of effective CDSSs through a meta-regression of 162 randomized trials [18]. Fung et al. compared three commercial knowledge bases used in CDSSs to detect drug-drug interactions [19]. In another study, Ratwani et al. analyzed user-centered design processes in EHRs from 11 vendors [20]. The analysis provided insights on how to improve these EHRs. In today’s healthcare system, hospitals and other healthcare centers might either purchase a commercial EHR system with or without a CDSS or opt to develop a homegrown (in-house) system. In either case, there has been many calls to improve commercial as well as homegrown EHR and CDSS systems since their inception [19, 21]. Scheife et al. convened experts and developed consensus recommendations for systematic evaluation of drug-drug interactions for CDSSs [21].

Currently, evidence for the benefits and desirable features of EHRs and CDSS is limited and contradictor. Similarly, little was narrated on which important safety features to consider when designing, developing, and/or upgrading EHRs with embedded CDSSs. As many hospitals and healthcare centers design, develop, and/or upgrade their own homegrown EHRs and CDSSs, healthcare providers and IT/programming experts are left wondering what safety features are important to consider in the absence of real guidelines helping developers and implementers of EHRs and CDSSs.

This study was conducted to develop a core list of important safety features and desiderata to consider when planning for, designing, developing, implementing, piloting, evaluating, maintaining, upgrading, and/or using EHRs with CDSSs and explore the opinions and views of a panel of experts in EHRs compared to handwritten patient records with regards to patient safety, cost, record keeping, and workflow. The core list of important safety features and desiderata might be used to guide decision makers in healthcare and IT/programming sectors when planning for, designing, developing, implementing, piloting, evaluating, maintaining, upgrading, and/or using EHRs with CDSSs.

## Methods

### Study context

In Palestine, healthcare is delivered through three main sectors: the government, the United Nations Relief and Works Agency for Palestine Refugees (UNRWA), and the private sector. Many hospitals in Palestine currently adopt and use some sort of EHRs. Recently, the Palestinian Ministry of Health has applied an electronic health information system (HIS) to connect all governmental hospitals operating in the West Bank of Palestine using AviCenna Health Information Medical System [22]. In 2009, the UNRWA started developing an EHR system for its 143 health centers in Jordan, Syria, Lebanon, the West Bank, and Gaza Strip. In 2017, the in-house built EHR system that was developed to improve services for common illnesses, maternal and child health, non-communicable diseases, laboratory and pharmacy was implemented in 121 health centers of which 100 were paperless [23].

Because different EHR systems are used in Palestinian hospitals, it is not always possible to efficiently transfer and/or share patient information between different healthcare delivery establishments. EHRs used in governmental hospitals contain features that are missing from EHRs used in the other sectors, and vice versa. Currently, there are many design, logistic, technical, and compatibility issues that need to be addressed before efficient transfer of patient information between different providers of healthcare services would become possible. Probably, the first step in addressing these issues is to achieve consensus on the important features that need to be considered when planning for, designing, developing, implementing, piloting, evaluating, maintaining, upgrading, and/or using EHRs with embedded CDSSs.

### Study design

**Fig. 1:**
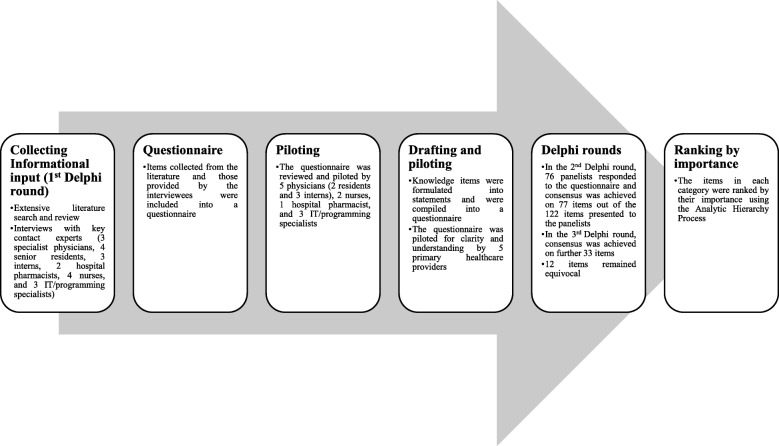
Flowchart illustrating the different phases of the study

In the current study, a mixed method combining the Delphi technique and the Analytic Hierarchy Process was used. The Delphi technique was used as a formal consensus technique to develop and achieve consensus on a core list of important safety features to be considered when planning for, designing, developing, implementing, piloting, evaluating, maintaining, upgrading, and/or using EHRs with CDSSs. The Analytic Hierarchy Process was used to rank these features (which will be referred to as items from now and on) in the order of their importance. Figure 1 shows a flowchart illustrating the different phases of the study.

### Collecting potentially important items

#### Literature search

A thorough literature search was conducted to identify potentially important items to be considered when planning for, designing, developing, implementing, piloting, evaluating, maintaining, upgrading, and/or using EHRs with CDSSs (Fig. 1). Although a systematic review of the literature was not performed in this study, whenever possible, PRISMA statement was followed [24]. Adherence to PRISMA statement is shown in Additional file 1.

The databases MEDLINE, EMBASE, COCHRANE, CInAHL, SCOPUS, and the search engine Google Scholar were used. The search strategy combined key terms like “computerized”, “electronic”, “patient”, “inpatient”, “medical”, “health”, “hospital”, “record”, “profile”, “chart”, “prescribing”, “e-prescribing”, “e-health”, “e-prescription”, “medicine”, “medication”, “clinical decision support”, “embedded”, “integrated”, “incorporated”, “coupled”, “features”, “entry”, “information”, “evaluation”, “safety”, “assessment”, “review”, and “quality”. The terms used were combined using “AND” and “OR” options. The search was supplemented with a manual search within the references of the articles retrieved from the search. Together, the manual search within the references of the articles retrieved and Google Scholar allowed inclusion of grey literature. The final literature search was performed on March 12th, 2019. Titles and abstracts of articles retrieved from the search were screened manually before deciding on which articles would be subjected to a full text review [1, 2, 4–7, 9, 10, 17–21, 25–62]. Original studies, reviews, and reports were included if they reported standard and advanced features of EHRs and CDSSs. Papers on prevalence and incidence of medical errors in health records were not included. As the main desired outcome of this search was to identify and obtain potentially important items to be considered, articles with mention of safety, features, and/or quality of EHRs with embedded CDSSs were given a priority for a full text review. Articles were reviewed if they were in English language. No restrictions to study design, year of publication, and publication status were applied.

Titles and abstracts of the retrieved papers and reports were screened for eligibility after removing duplicate results. The literature search was conducted by the main investigator (PhD). To ensure reproducibility, the screening process was repeated three times. The literature search was verified by another researcher (PhD) who had previous experience in conducting literature search within the databases used. The second researcher reviewed all the decisions to include and/or exclude titles and noted any discrepancies. Conflicting results were resolved by discussion and consensus.

#### Extraction of items

The full text of each paper and report included was reviewed by the main author (PhD). A data collection form was created using an Excel spreadsheet (Microsoft Inc.) which was used to collect the items extracted by the main investigator (PhD) from the literature. To ensure reproducibility, the process was repeated three times. Another researcher (PhD) independently reviewed the highlighted text and the items extracted into the data collection form. Conflicts were resolved by discussion and consensus. A list of items was constructed after removing duplicates.

### The first Delphi round: interviews with key contact experts

Key contact experts in the field were interviewed to collect more potential items that were not found in the literature with the objective of supplementing the list of items extracted from the literature. Whenever possible, consolidated criteria for reporting qualitative research (COREQ) were followed [63]. Adherence to COREQ checklist is shown in Additional file 2.

Potential participants were identified through personal contacts in the field. A purposive sampling method was used to invite and recruit the study participants. An email was sent to 25 potential participants inviting them to take part in the study. The email invitation included details of the design and objectives of the study. The sample size was informed by previous studies in which interviews were conducted to supplement items to be used in the subsequent iterative Delphi rounds [8, 22, 64–68]. Those who did not respond receive 3 reminders, each reminder was 1 week a part. Those who were willing to participate replied with an email expressing their interest in participation in the study. Prior to interviews, the interviewees were informed that the study was being conducted as a research study with the far aim of improving EHRs and CDSSs in Palestinian hospitals. The interviewees were also informed that investigators had no personal interest to influence the final outcomes of the project. The interviews were conducted by the main investigator (PhD) who was male in gender and had previous experience in conducting interviews and studies using the Delphi technique. The interviewer held an academic associate professor position at the time of the interviews. The key contacts were extensively involved either in planning for, designing, developing, implementing, piloting, evaluating, maintaining, upgrading, and/or using EHRs with CDSSs for a period of not less than 5 years. The participants were interviewed one-on-one in a calm side of their workplaces. After obtaining their sociodemographic and practice/experience details, the interviewees were asked to mention all items that were, in their opinion, important to be considered when planning for, designing, developing, implementing, piloting, evaluating, maintaining, upgrading, and/or using EHRs with CDSSs. The interviews were open and on occasions, the interviewer mentioned a feature that was provided by another interviewee. The objective of providing such prompts was to provide the interviewee an opportunity to “hitch-hike” with other interviewees and mention more potential items. The interviews were audio-recorded and were not repeated. On occasions, the interviewer made notes of items mentioned by the interviewees during the interviews. The main investigator listened to each recording three times and the interviews were analyzed for their content. Items were interpreted from the recordings and extracted directly into the data collection form. Analysis was repeated again and continued till all items were grouped. Grouping was derived from the data. Items obtained from each interview were returned to the interviewee for corrections and if they wished to add more items. Participants were recruited from 5 different Palestinian hospitals and IT/programming companies.

Together, items collected from the literature and those mentioned by the interviewees were summarized and formulated into statements. These statements were compiled into an initial list of potentially important items which was then used as input in the subsequent iterative Delphi rounds.

### The questionnaire

A questionnaire was designed to contain 3 sections. The first section was destined to collect the sociodemographic, academic, and professional variables of the participants. In this section, the participants (panelists) needed to disclose their gender, age, academic qualifications, specialty/rank/hierarchy, and employer. The second part was destined to explore the views and opinions of the panelists on their perceived impact of EHRs with embedded CDSSs compared to hand-written paper-based patient records. In this section, the panelists were supposed to express the level of their disagreement/agreement on 12 items related to patient’s safety, costs, record keeping, and workflow using a Likert-scale of 1–5 (1 indicated strong disagreement and 5 indicated strong agreement). The 5-point Likert-scale was extensively used in measuring the level of disagreement/agreement on items presented to participants [69]. A space was left for comments and the panelists were encouraged to include written qualitative comments to supplement their scores. The third section contained the initial list of potentially important items to be presented in the Delphi iterative rounds. The list contained 122 items that were collected from the literature and/or provided by the interviewees during the interviews. The panelists had to express the level of their disagreement/agreement with each potentially important item using a Likert-scale of 1–9 (1 indicated strong disagreement, i.e. in the participant’s view, the item was not important and 9 indicated strong agreement, i.e. in the panelist’s view, the item was important). Although many consensus indices have been used in Delphi technique [70], the 9-point Likert-scale has emerged as one of the most commonly employed scale in panelists’ extent of disagreement/agreement with items in the Delphi technique [8, 22, 64–67]. Below each item, a space was provided and the panelists were encouraged to write comments to qualify/justify their scores. As a high response rate was desired in this study, the panelists received paper-based questionnaire by hand.

### Reviewing and piloting the questionnaire

The questionnaire was reviewed and piloted by 5 physicians (2 residents and 3 interns), 2 nurses, 1 hospital pharmacist, and 3 IT/programming specialists (Fig. 1). The aim of the review and pilot was to test the questionnaire for readability and comprehensibility. Based on comments of the participants, some items were rephrased for clarity and some medical and programming specific terms (jargon) were replaced by simpler terms.

### Panel of healthcare providers and IT/programming specialists

A panel of healthcare providers (physicians, nurses, and hospital pharmacists) and IT/programming specialists was recruited using a purposive sampling technique (Fig. 1). The potential panelists were identified and approached using personal contacts in the field. Selection of the panelists is one of the most critical steps in the Delphi technique. The panelists need to be knowledgeable of the subject being investigated [71]. In this study, the panelists selected were rich in information and had appreciable experience in planning for, designing, developing, implementing, piloting, evaluating, maintaining, upgrading, and/or using EHRs with CDSSs. The panelists were selected, approached, invited, and included based on their existing experience and involvement in planning for, designing, developing, implementing, piloting, evaluating, maintaining, upgrading, and/or using EHRs with CDSSs. The selection process ensured diversity of the panelists in terms of gender, age group, profession, academic qualifications, length of working experience, rank/hierarchy, and type of employer. Upon invitation, the design and objectives of the study were explain to potential panelists. The panelists needed to provide their informed consent to qualify for this study. The inclusion criteria were as follows: 1) possessing a qualifying academic degree in one of the healthcare or IT/programming professions, 2) having a license to practice (for healthcare providers), 3) at least 5 years of experience in using or involvement in planning for, designing, developing, implementing, piloting, evaluating, maintaining, upgrading, and/or using EHRs with CDSSs, 4) employment in a public or private hospital, or IT/programming firm involved in planning for, designing, developing, implementing, piloting, evaluating, maintaining, upgrading, and/or using EHRs with CDSSs, and 5) willingness to participate and providing informed consent. Physicians who were specialists/consultants, residents, and interns were recruited to the panel. Senior and staff hospital pharmacists and nurses were also recruited. Recruitment of hospital pharmacists and nurses was intentional to ensure representation of other healthcare provision team. IT/programming specialists were also represented in the panel. The panel included 76 panelists. The number of panelists recruited for this study was within the range of number of panelists used in previous studies in which the Delphi technique was used to develop and achieve formal consensus on issues lacking consensus in healthcare [22, 41, 64–68, 72–81]. It is noteworthy mentioning that currently there is no formal consensus on the number of panelists to be included in a study using the Delphi technique. Previous studies used panel sizes in the range of 10 to 1000 [78]. No financial incentives were offered to the panelists in exchange of their participation in this study.

### The iterative Delphi process

All panelists received paper-based copies of the questionnaire and an iterative Delphi process with controlled feedback was initiated (Fig. 1). In the second Delphi round, the panelists provided their sociodemographic, academic, and professional details, expressed their level of their disagreement/agreement on the 12 statements, and voted on each of the 122 items. The Delphi technique was conducted in accordance with the conducting and reporting of Delphi studies (CREDES) guidelines [81]. Adherence to CREDES guidelines is shown in Additional file 3.

### Analysis of votes

Data were extracted from the questionnaires completed in the second Delphi round and were entered into an Excel Sheet (Microsoft Inc.). Basic descriptive statistics like the 1st quartile (Q1), median (Q2), 3rd quartile (Q3), and the interquartile range (IQR). The IQR was computed by subtracting (Q3 - Q1). The descriptive statistics were computed for each item separately. The qualitative comments were analyzed for their content, summarized, and categorized by the main investigator (PhD). Summaries were returned to the participants for feedback and corrections.

### Definition of consensus

The definition of consensus used in this study was informed by previous studies in which the Delphi technique was used to achieve formal consensus on issues in healthcare [8, 22, 64–68, 74, 82]. Briefly, when a panelist scored a vote of 1–3 on an item, this meant that in the opinion of the panelist, the item was not important and should be excluded from the final core list. When a panelist scored a vote of 7–9 on an item, this meant that in the opinion of the panelist, the item was important and should be included in the final core list. When a panelist scored a vote of 4–6 on an item, this meant that the panelist was indecisive if the item was important or not (partial agreement). Criteria for consensus were defined a priori as: 1) when the median of scores on an item was within the range of 1–3 and the IQR was ≤2 of at least 75% of the panelists, the item was considered as unimportant and was excluded from the final core list, 2) when the median of scores on an item was within the range of 7–9 and the IQR was ≤2 of at least 75% of the panelists, the item was considered as important and was included in the final core list, and 3) when the median of scores on an item was within the range 4–5 and/or the IQR was > 2, the item was considered equivocal. It was decided a priori that all equivocal items in the second Delphi round would be subjected to a third Delphi round.

### The revised questionnaire containing equivocal items and the third iterative Delphi round

All items that remained equivocal in the second Delphi round were included into a revised questionnaire (Fig. 1). For each equivocal item, the panelists were provided with: 1) a reminder of their own score, 2) the median score, 3) the IQR, and 4) summary of the qualitative comments that were made by other panelists to qualify/justify their scores at the time of voting. The panelists were given the chance to reconsider or maintain their scores in view of the scores and comments of the other panelists. Anonymity of the panelists was maintained during the Delphi rounds. Scores obtained in the third Delphi round were analyzed using the same definitions used in the second Delphi round.

Informed by previous studies in which the Delphi technique was used to achieve consensus on issues in healthcare and based on the votes and comments of the panelists in the third Delphi round, consensus on the remaining equivocal items was unlikely. Therefore, it was decided not to conduct a fourth Delphi round.

Items on which consensus was achieved to be included into the final core list were grouped by the main investigator (PhD) into 9 categories. Groups were derived from the items on which consensus was achieved to be included in the final core list.

### Ranking of items in order of their importance

In this study, it was decided to rank the items that were included in the final core list in order of their importance using the Analytic Hierarchy Process (Fig. 1). The Analytic Hierarchy Process is a powerful tool that has been extensively used in multi-criteria decision analysis, notably, in healthcare [83–85]. The Analytic Hierarchy Process allows addressing and facilitating making decisions on complex problems with multi-criteria. The method is especially appropriate for small group settings [83, 85]. Using this method, participants can make pairwise comparisons in order to hierarchize items in the order of their perceived importance.

Ten participants who already participated in the Delphi rounds were purposively approached and invited to take part in this step. Participants who provided more qualitative comments on the items during the Delphi rounds to qualify/justify their scores were given a priority. The participants were asked to make pairwise comparisons of items within the same category using a scale of 9-points. When a higher score was given to an item, this indicated the higher importance of that item compared to the other items in the same category. Scores from each participant were used to create matrices in Excel Spreadsheets. Mathematical formulas originally developed by Saaty were used to calculate importance weights (%) with their consistency ratios [86]. Scores were considered when their consistency ratios were less than 0.1. For each category, importance weights (%) were calculated for each item within all categories. Importance weights (%) of all items within the same category sum to 100%.

### Statistical analysis

Scores on each item were entered into GraphPad Prism 6.0 for Windows (GraphPad Software). One-way analysis of variance (ANOVA) with Bonferroni post-hoc tests were used to compare differences in importance weights (%) [85]. Statistical significance was considered * when the *p*-value was < 0.05, ** when the *p*-value was < 0.01, *** when the *p*-value was < 0.001, and **** when the *p*-value was < 0.0001.

### Ethical considerations

This study was approved by the Institutional Review Board (IRB) of An-Najah National University. The panelists understood that the Delphi technique was a semi-anonymous technique in which the identity of the panelist is known to the investigator while the panelist remains anonymous to the rest of the panel members. Scores of the panelists weighed equally in the analysis. All participants provided written informed consent.

## Results

### Search results

The literature search yielded a total of 4023 records. Duplicate records were removed. The rest of records were screened for ineligibility based on title and abstract, data were extracted from 51 records.

### Study participants

#### Interviews

Interview invitations were sent to 25 key contact experts. Of those, 21 (84.0%) expressed interest in participation and 4 (16.0%) were not available at that time. Interviews could not be scheduled with 2 (8.0% of those originally invited) who later declined due to personal reasons and time constrains. Interviews were conducted with 19 (76.0% of those originally invited) key contacts in the field who were 3 specialist physicians, 4 senior residents, 3 interns, 2 hospital pharmacists, 4 nurses, and 3 IT/programming specialists. Respondents belonged to all specialties that were purposively invited in the study. The detailed sociodemographic, academic, and professional details of the panelists who participated in this stage of the study are shown in Table 1. The median duration of the interviews was 37 with an IQR of 16 min.

**Table 1:** Sociodemographic, academic, and professional details of the panelists who participated in the study (*n = 76*)

	Interviewees (*n* = 19)		Delphi rounds (*n* = 76)		Analytic Hierarchy Process (*n* = 10)	
Variable	*n*	%	*n*	%	*n*	%
Gender						
Male	13	68.4	45	59.2	6	60.0
Female	6	31.6	31	40.8	4	40.0
Age (years)						
< 40	3	15.8	34	44.7	2	20.0
≥ 40	16	84.2	42	55.3	8	80.0
Academic qualifications						
BSc	5	26.3	16	21.1	2	20.0
MSc	2	10.5	13	17.1	2	20.0
PhD	2	10.5	9	11.8	1	10.0
MD	6	31.6	28	36.8	3	30.0
Pharm.D	1	5.3	4	5.3	–	–
MD/PhD	3	15.8	6	7.9	2	20.0
Specialty/rank/hierarchy						
Physician	**10**	**52.6**	**32**	**42.1**	**5**	**50.0**
Intern	3	15.8	5	6.6	1	10.0
Resident	4	21.1	11	14.5	2	20.0
Specialist/consultant (internists)	3	15.8	16	21.1	2	20.0
Hospital pharmacists	**2**	**10.5**	**11**	**14.5**	**1**	**10.0**
Staff (< 10 years)	1	5.3	5	6.6	–	–
Senior (≥ 10 years)	1	5.3	6	7.9	1	10.0
Nurse	**4**	**21.1**	**14**	**18.4**	**1**	**10.0**
Staff (< 10 years)	1	5.3	6	7.9	–	–
Senior (≥ 10 years)	3	15.8	8	10.5	1	10.0
IT/Programming	**3**	**15.8**	**19**	**25.0**	**3**	**30.0**
Junior (< 10 years)	1	5.3	8	10.5	1	10.0
Senior (≥ 10 years)	2	10.5	11	14.5	2	20.0
Employer						0.0
Public hospital	9	47.4	42	55.3	4	40.0
Private hospital	7	36.8	18	23.7	3	30.0
IT/Programming sector	3	15.8	16	21.1	3	30.0

*BSc* Bachelor of Science, *IT* information technology, *MD* Doctor of Medicine, *MSc* Master of Science, *Pharm.D* Doctor of Pharmacy, *PhD* Doctor of Philosophy

#### The panelists

In this study, a total of 76 panelists who were invited voted in the second and third iterative Delphi rounds, giving a response rate of 100%. Both genders were represented in the panel, females represented 40.8% of the panel members. The panel members belonged to different age groups (55.3% were 40 years old and above). Of the participants, the largest groups were physicians (42.0%) and IT/programming specialists (25.0%). However, nurses and hospital pharmacists were also represented in the panel. The panelists were employed by private and public hospital, as well as, IT/programming firms. The detailed sociodemographic, academic, and professional details of the panelists who participated in this stage of the study are shown in Table 1.

#### The analytic hierarchy process

A total of 10 participants made pairwise comparisons of the items in the Analytic Hierarchy Process. All 10 participants who were invited to take part in this step made pairwise comparisons, giving a response rate of 100%. The participants were physicians, pharmacists, nurses, and IT/programming specialists. Of all participants, 50.0% were physicians by profession. The detailed sociodemographic, academic, and professional details of the panelists who participated in this stage of the study are shown in Table 1.

### Merits of EHRs with embedded CDSSs compared to traditional paper-based handwritten patient records

#### Merits related to patient’s safety

The vast majority (93.4%) of the panelists agreed (either strongly agreed or agreed) that EHRs with embedded CDSSs can reduce medication errors compared to traditional paper-based handwritten patient records (Table 2). However, those who voted neutral or with disagreement commented that EHRs with embedded CDSSs were associated with other types of errors. Of all panelists, 77.6% agreed that EHRs with embedded CDSSs can reduce adverse medication events. Those who disagreed or were neutral cited conflicting findings reported in the literature. The vast majority of the panelists (89.4%) agreed that EHRs with embedded CDSSs can improve communication between healthcare providers. Those who disagreed or were neutral stated that more interactions were needed to resolve issues when inaccurate information was entered. About 80% of the panelists agreed that EHRs with embedded CDSSs can improve the overall patient care experience. Those who did not agree cited poor design of systems, drop-down menus, screen, and automatic filling functions might increase likelihood of errors and may negatively impact the overall patient care experience.

**Table 2:** Views and opinions of the panelists on EHRs with embedded CDSSs

	Item	*n*	%	Qualitative comments	Source of the xitem
	Compared to paper-based handwritten patient records, EHRs with embedded CDSSs can:				
Patient safety	**1. Reduce medication errors**				B
	Strongly agree	53	69.7	EHRs might be associated with other types of errors like omissions and entry of inaccurate information	
	Agree	18	23.7		
	Neutral	3	3.9		
	Disagree	1	1.3		
	Strongly disagree	1	1.3		
	**2. Reduce adverse reactions**				B
	Strongly agree	26	34.2	Evidence for reducing adverse medication reactions is mixed	
	Agree	33	43.4		
	Neutral	12	15.8		
	Disagree	3	3.9		
	Strongly disagree	2	2.6		
	**3. Improve communication between healthcare providers**				B
	Strongly agree	27	35.5	In case of inaccurate information, more interactions are needed for corrective actions	
	Agree	41	53.9		
	Neutral	6	7.9		
	Disagree	1	1.3		
	Strongly disagree	1	1.3		
	**4. Improve overall patient care experience**				B
	Strongly agree	36	47.4	Poor design of systems, drop-down menus, screen design, and automatic filling functions might increase likelihood of errors and may negatively impact the overall patient care experience	
	Agree	25	32.9		
	Neutral	12	15.8		
	Disagree	2	2.6		
	Strongly disagree	1	1.3		
Cost	**5. Improve prescriber’s ability to prescribe more cost-effective medications**				I
	Strongly agree	18	23.7	This might be dependent on the list of product options available in the system	
	Agree	34	44.7		
	Neutral	18	23.7		
	Disagree	4	5.3		
	Strongly disagree	2	2.6		
	**6. Increase prescriber’s likelihood to discontinue unnecessary and/or ineffective medications**				B
	Strongly agree	14	18.4	Evidence for reducing ineffective medications is mixed	
	Agree	42	55.3		
	Neutral	15	19.7		
	Disagree	2	2.6		
	Strongly disagree	3	3.9		
	**7. Decrease costs associated with adverse medication reactions and medication errors**				I
	Strongly agree	51	67.1	EHRs are associated with other types of errors	
	Agree	16	21.1		
	Neutral	6	7.9		
	Disagree	2	2.6		
	Strongly disagree	1	1.3		
Record keeping	**8. Improve storage of patient information and prescription records**				L
	Strongly agree	53	69.7	More efforts are needed to keep and maintain copies of these electronic copies	
	Agree	21	27.6		
	Neutral	2	2.6		
	Disagree	0	0		
	Strongly disagree	0	0		
	**9. Improve prescriber’s ability to trace patient’s prescribing information**				B
	Strongly agree	50	65.8	More efforts are needed to improve search and retrieval activities	
	Agree	23	30.3		
	Neutral	3	3.9		
	Disagree	0	0		
	Strongly disagree	0	0		
	**10. Improve prescriber’s ability to monitor medications and evaluate clinical outcomes**				L
	Strongly agree	31	40.8	More efforts are needed to improve reminders that should pop-up only when necessary to avoid prescriber’s desensitization	
	Agree	26	34.2		
	Neutral	15	19.7		
	Disagree	2	2.6		
	Strongly disagree	2	2.6		
Workflow	**11. Reduce wait times needed to call prescribers to clarify illegible or ambiguous medication orders**				B
	Strongly agree	38	50	In case of technology failures and system malfunctions, more wait times are needed	
	Agree	27	35.5		
	Neutral	9	11.8		
	Disagree	1	1.3		
	Strongly disagree	1	1.3		
	**12. Improve workflow of prescribers and other healthcare team by enabling copying and editing of prescribing information**				L
	Strongly agree	14	18.4	Workflow challenges, more personnel, training, and maintenance efforts are needed	
	Agree	22	28.9		
	Neutral	17	22.4		
	Disagree	12	15.8		
	Strongly disagree	11	14.5		

*B* both (literature and interviews), *EHRs* electronic health records, *CDSSs* clinical decisions support systems, *I* interviews, *L* literature

#### Merits related to costs

About 68% of the panelists agreed that EHRs with embedded CDSSs can improve the prescriber’s ability prescribe more cost-effective medications (Table 2). The panelists who did not agree stated that this might depend on the list of product options available through the system for the prescriber to choose from. About 74% of the panelists agreed that EHRs with embedded CDSSs can increase the prescriber’s likelihood to discontinue unnecessary and/or ineffective medications. Those who did not agree stated that the literature was inconclusive. About 88% of the panelists agreed that EHRs with embedded CDSSs can decrease the costs associated with adverse medication events and medication errors. Those who did not agree stated that there were other costs associated with the other types of errors encountered in EHRs with embedded CDSSs.

#### Merits related to record keeping

The vast majority of the panelists (97.3%) agreed that EHRs with embedded CDSSs can improve storage of patient information and prescription records (Table 2). Those who did not agree cited efforts needed to keep and maintain copies of these EHRs. Again, the vast majority (about 96%) of the panelists agreed that EHRs with embedded CDSSs can improve prescriber’s ability to trace patient’s prescribing information. Those who did not agree cited efforts needed to improve search and retrieval through such systems. 75% of the panelists agreed that EHRs with embedded CDSSs can improve prescriber’s ability to monitor medications and evaluate clinical outcomes. Those who did not agree cited efforts needed to improve reminders that should pop-up only when necessary to avoid prescriber’s desensitization.

#### Merits related to workflow

About 86% of the panelists agreed that EHRs with embedded CDSSs can reduce wait times needed to call prescribers to clarify illegible or ambiguous medication orders (Table 2). Those who did not agree mentioned that more wait times were needed when technology failed and systems malfunctioned. However, only about 47% of the panelists agreed that EHRs with embedded CDSSs can improve workflow of prescribers and other healthcare team by enabling copying and editing of prescribing information. However, more than half of the panelists did not agree and cited challenges to workflow, more personnel, training, and maintenance efforts needed.

### The iterative Delphi rounds

#### Items included in the core list with their weights of importance

Of the 122 items presented to the panelists in the second Delphi round, consensus was achieved to include 77 (63.1%) items. The remaining 45 items were included into the revised questionnaire and were subjected to a third Delphi round. In the third Delphi round, consensus was achieved on further 33 (27.1%) items. Following the two iterative Delphi rounds, consensus was achieved on 110 (90.2%) items. These items were grouped under 9 categories. Of these, 16 (14.5%) items were related to the demographic characteristics of the patient, 16 (14.5%) were related to prescribing medications, 16 (14.5%) were related to checking prescriptions and alerts, 14 (12.7%) items were related to the patient’s identity, 13 (11.8%) items were related to patient assessment, 12 (10.9%) items were related to the quality of alerts, 11 (10%) items were related to admission and discharge of the patient, 9 (8.2%) items were general features, and 3 (2.7%) items were related to diseases and making diagnosis. All categories and items ranked in the order of their importance weights are listed in Table 3. The Additional file 4:Tables S1-S9 show multiple comparisons of importance weights items in each category.

**Table 3:** Important features of EHRs with embedded CDSSs on which consensus was achieved in this study

		Round 02			Round 03			Importance weight (%)		Source of the item
#	Items	M	IQR	%A	M	IQR	%A	M	SD	
Demographic characteristics of the patient										
1	The body weight of the patient	8	2	88	NA			11.2	3.8	B
2	The measure units of weight (gm, kg, pounds)	8	2	92	NA			10.6	2.9	L
3	The working weight of the patient that was used for dose calculations (for example, 5 kg instead of 5.1 kg)	7	2	84	NA			9.3	3.0	L
4	Date on which the weight of the patient was measured	7	2	79	NA			8.6	3.2	B
5	The height of the patient	6	5	53	8	2	75	8.1	2.8	B
6	The measure units for height (cm, m, in)	5	4	51	7	2	76	7.6	2.4	L
7	The date on which the height of the patient was measured	4	4	48	7	2	75	7.2	2.5	B
8	The body surface area of the pediatric patient	7	2	79	NA			6.8	2.3	L
9	The measure units of the body surface area	7	2	82	NA			6.0	2.1	L
10	The date on which the body surface area was measured	7	1	85	NA			5.4	3.1	L
11	The body mass index of the patient	7	2	76	NA			4.8	3.2	L
12	The date on which the body mass index of the patient was measured	7	2	75	NA			3.9	2.9	L
13	Time on which the weight of the patient was measured	7	4	71	7	2	82	2.9	2.6	L
14	The time on which the height of the patient was measured	4	5	46	7	2	76	2.7	2.4	L
15	The time on which the body surface area was measured	7	2	84	NA			2.6	2.0	L
16	The time on which the body mass index of the patient was measured	6	4	61	7	2	75	2.3	1.7	L
Prescribing medications										
1	Prompting a mode of selection for specifying the dose (for example mg, μg, mL, … etc.) of the medication prescribed	9	1	100	NA			11.8	2.6	B
2	Prompting a mode of selection for specifying the frequency (number of times) the medication needs to be administered (for example, once daily, twice daily, three times daily, ...etc.)	9	1	100	NA			9.4	3.2	B
3	Prompting a mode of selection for specifying the route by which the medication would be administered (for example, oral, intravenous, intramuscular, … etc.)	8	1	99	NA			8.8	3.6	B
4	Prompting a mode of selection for specifying the dosage form “formulation” (for example, tablet, capsule, syrup, ...etc.) of the medication prescribed	8	2	91	NA			8.2	2.9	B
5	Prompting a mode of selection for specifying the number of dosing units to be administered each time (for example, one tablet, two tablets, … etc.)	9	1	93	NA			7.5	2.6	L
6	Allowing search and/or providing a mode of selection (for example a drop-down menu) for all medications available on the hospital’s formulary including their non-proprietary names and brand (branded-generic) names	7	2	79	NA			6.9	2.4	L
7	Prompting a mode of selection for specifying the date on which the medication was prescribed	9	1	95	NA			6.4	2.1	L
8	Prompting a mode of selection for specifying the duration for which the medication administration should be continued	8	1	97	NA			6.2	2.8	L
9	Prompting a mode of selection for specifying the date on which the medication administration should be started	7	2	93	NA			5.8	2.7	L
10	Prompting a mode of selection for specifying the times at which the medication doses should be administered (for example, at 8:00 am, 2:00 pm, … etc.)	8	1	92	NA			5.3	2.4	L
11	Prompting a mode of selection for specifying the date on which the medication administration should be discontinued	9	1	92	NA			5.1	1.9	B
12	Prompting a mode of selection for specifying the time on which the medication administration should be discontinued	9	1	94	NA			4.8	1.8	L
13	Prompting a mode of selection for specifying the medication administration in relation to meals	8	1	92	NA			4.3	1.6	B
14	Prompting a mode of selection for specifying the maximal number of doses to be administered in 24 h for medication prescribed as “when needed” (PRN)	8	1	89	NA			3.8	1.2	B
15	Prompting a mode of selection for specifying the name of the physician who prescribed the medication	9	1	96	NA			3.2	1.1	L
16	Ability to suggest other suitable substitutes (other medications from the same pharmacological class)	6	4	54	7	2	76	2.5	1.4	I
Checking prescriptions and alerts										
1	Ability to assess suitability of the dose in view of the patient’s conditions like renal and/or hepatic functions	8	1	92	NA			13.6	3.6	B
2	Clear instructions to guide prescribers on the procedures to follow when a medication order to be discontinued or changed	9	2	92	NA			12.8	3.1	B
3	Ability to check for and provide warnings on potential drug-drug interactions	8	2	92	NA			11.3	3.5	L
4	Ability to check for and provide warnings on potentially contraindicated medications for the patient	7	2	89	NA			10.2	3.2	B
5	Ability to check for and provide warnings on potential drug-food interactions	5	3	61	7	2	75	9.6	3.3	L
6	Ability to check for and provide warnings on potential drug-herb interactions	6	3	68	7	1	78	8.5	2.9	L
7	Ability to provide warnings regarding any potential medication adverse reactions in view of the patient’s conditions	6	3	59	7	2	76	7.3	2.6	B
8	Ability to recommend evidence-based dose suitable for the patient	7	3	69	7	1	79	6.4	2.8	B
9	Ability to check for and provide warning when another medication from the same pharmacological class (duplication) is prescribed	7	4	69	7	2	76	5.2	2.9	L
10	Ability to provide prompts on special precautions or procedures to administer the prescribed medication (if any)	7	3	74	7	2	77	3.8	2.3	L
11	Ability to alert the prescriber if the dosage form(s) prescribed was (were) of slow or modified release	6	3	69	7	2	76	2.6	1.8	L
12	Ability to enter reason(s) (justification) why another medication from the same pharmacological class (duplication) is prescribed	7	2	79	NA			2.4	1.6	L
13	Ability to add reasons (justification) for not changing the medication or dose in the event of an adverse medication reaction	5	3	46	7	2	75	2.1	1.9	L
14	Ability to enter reason(s) (justification) why the dose was different from the evidence-based recommended one	7	2	78	NA			1.7	1.2	L
15	Ability to enter reason(s) (justification) why the dosing frequency was different from the evidence-based recommended one	6	4	58	7	2	75	1.4	1.3	L
16	Ability to enter reason(s) (justification) why the duration of medication administration was different from the evidence-based recommended one	5	4	56	7	2	76	1.1	0.9	L
Patient’s identity										
1	The first name of the patient	9	1	100	NA			12.2	3.7	B
2	The father’s name of the patient	9	1	100	NA			11.3	3.2	B
3	The grandfather’s name of the patient	9	1	100	NA			10.7	4.1	B
4	The family name (surname) of the patient	9	1	100	NA			9.2	3.6	B
5	The unique national identification number of the patient	6	4	62	7	2	76	8.7	2.9	B
6	The gender of the patient	9	1	100	NA			8.1	3.1	B
7	The date of birth of the patient	9	1	100	NA			7.5	2.9	B
8	The age of the patient	7	2	89	NA			7.1	3.1	L
9	The measure units of age (years, months, or days)	8	2	91	NA			6.8	2.8	L
10	The gestational age of the pediatric patient (for neonates)	7	4	71	7	2	77	6.1	1.9	L
11	The corrected gestational age of the pediatric patient (if the neonate was a preterm)	6	4	68	7	2	76	5.3	2.8	L
12	The date on which the age of the patient was calculated	7	2	88	NA			3.5	2.6	L
13	The telephone number of the patient/their parent(s)/guardian(s) in case of a pediatric patient	7	3	74	7	2	79	2.1	1.2	B
14	The home address of the patient	7	3	72	7	2	78	1.4	1.1	B
Patient assessment										
1	Prompts to enter the presenting symptoms of the patient	8	1	99	NA			12.6	3.2	B
2	Prompts to enter the vital signs of the patient	9	1	99	NA			11.3	3.5	B
3	Ability to enter and/or automatically import results of laboratory tests ordered for the patient	9	1	99	NA			10.2	2.8	L
4	Ability to enter and/or automatically import results of medical images ordered for the patient	9	1	99	NA			9.3	2.6	L
5	Ability to enter other co-morbidities the patient might be suffering from	8	1	97	NA			8.9	2.3	B
6	Ability to enter all relevant information on prescription medications the patient is/was taking	8	1	96	NA			8.2	2.9	B
7	Ability to enter all relevant information on other non-prescription medications the patient is/was taking	8	2	94	NA			7.3	3.1	I
8	Ability to enter all relevant information on allergies to medications the patient suffered from	9	1	100	NA			7.0	2.2	B
9	Ability to enter all relevant information on adverse medication reactions the patient suffered from	9	1	100	NA			6.3	1.9	L
10	Ability to update patient’s data and integrating new laboratory, imaging, and vital sign measurements	9	1	98	NA			6.0	1.8	L
11	Ability to transfer patient’s data into the patient’s electronic medical record	8	1	96	NA			4.8	2.0	L
12	Ability to enter information on congenital defects of the patient	8	1	94	NA			4.6	1.8	I
13	Ability to enter all relevant information on herbal medicines used by the patient	7	3	74	7	2	81	3.5	1.6	L
Quality of alerts										
1	Suggestions and alerts should be evidence-based, provide a reference or references, and level of evidence	6	4	52	7	2	75	16.9	3.2	L
2	Suggesting evidence-based and up-to-date recommendations, guidelines, and/or protocols to prescribe medications	6	3	74	7	2	79	14.2	2.6	B
3	Alerts regarding allergy should distinguish between a serious potential allergy and minor side effect of the medication	7	2	76	NA			12.9	3.8	L
4	Alerts and suggestions should provide clear information on relative risk of harm for the given patient	6	4	51	7	2	76	11.3	3.5	L
5	Ability to give warning when the prescribed dose differed from the recommended dose	6	4	61	7	2	76	8.6	2.9	B
6	Ability to recommend evidence-based dosing frequency suitable for the patient	7	4	62	7	2	76	7.3	3.1	B
7	Ability to recommend evidence-based duration of medication administration	7	3	60	7	1	75	6.8	2.8	L
8	Clear instructions to guide prescribers on writing the reason for discontinuing or changing a medication order	8	2	87	NA			5.3	3.1	L
9	Prompts to indicate if additional charts other than the medication chart was used for the patient (for example other charts for intravenous fluids, nutrition, … etc.)	8	1	89	NA			4.8	2.1	B
10	Clear instructions to obtain parent/guardian authorization to allow for immunization as per the national program, in case, immunization was due for a pediatric patient	7	4	71	7	1	82	4.3	1.8	L
11	The system should not allow the use of non-standard abbreviations/nomenclature	8	2	94	NA			4.1	1.6	B
12	Compulsory review of medications prescribed before saving and validating orders	7	3	73	7	1	85	3.5	1.8	L
Admission and discharge of the patient										
1	The hospital’s admission number assigned to the patient at the time of admission	7	2	90	NA			20.2	2.3	B
2	The date on which the patient was admitted to the hospital	8	2	97	NA			15.8	2.1	B
3	Name(s) of the ward(s) to which the patient was (were) admitted	9	2	94	NA			13.1	3.2	B
4	The name of the physician under whose care the patient was admitted to the hospital	8	2	94	NA			11.9	4.1	L
5	The date on which the patient was discharged from the hospital	8	2	93	NA			11.1	2.6	B
6	The name of the physician who decided to discharge the patient	9	1	95	NA			9.1	3.1	B
7	Bed(s) number(s) that was (were) assigned to the patient during their admission to the hospital	9	1	98	NA			8.0	2.9	B
8	Name of the hospital to which the patient was admitted	9	2	93	NA			4.9	2.5	I
9	Name of the physician who entered the patient information and verified that all details were correct	7	2	88	NA			2.9	2.1	L
10	The time on which the patient was discharged from the hospital	7	2	91	NA			1.9	1.7	B
11	The time on which the patient was admitted to the hospital	7	2	93	NA			1.1	1.2	B
General features										
1	The system should be as user friendly as practically possible providing easy to use interfaces	9	1	96	NA			21.1	4.2	I
2	Alerts should be clear and specify exactly why they were displayed	8	2	91	NA			17.3	3.6	B
3	The system should provide a prepackaged entry forms allowing accurate and comprehensive patient assessment	8	2	94	NA			15.6	4.3	L
4	The system should allow retrieval and viewing of all and/or selected patient’s specific information as the user desires	7	2	88	NA			13.1	3.9	B
5	Users should provide reasons when opting to over-ride system recommendations	7	2	88	NA			10.2	2.8	L
6	Provided entries should be customizable in case the user needed to modify some of them	7	2	88	NA			8.6	2.7	B
7	Ability to remind the user to complete tasks and activities that were not completed or selected for follow up	7	2	77	NA			5.6	2.1	B
8	Users should be able to decline suggested recommendations	6	4	63	7	2	78	4.9	1.9	I
9	Alerts and suggestions should pop-up when really necessary to avoid prescriber alert desensitization	6	3	48	7	1	77	3.6	1.7	B
Diseases and making diagnosis										
1	Ability to enter diagnosis	8	1	98	NA			39.7	10.3	B
2	Ability to access to offline, online, and searchable databases and references related to diseases and differential diagnosis	8	2	91	NA			32.1	6.2	B
3	Ability to provide hints for potential diagnosis based on the data entered into the assessment section	7	2	87	NA			28.2	4.6	L

*%A* percentage of panelists who voted 7–9 on the item, *B* both (literature and interviews), *CDSSs* clinical decisions support systems, *EHRs* electronic health records, *I* interviews, *IQR* interquartile range, *L* literature, *M* median, *NA* not applicable, *SD* standard deviation

#### Items related to the demographic characteristics of the patient

The 16 items related to demographic characteristics of the patient on which consensus was achieve included body weight, height, surface area, and body mass index. Items in this category ranked by their importance weights are shown in Table 3. The item related to the body weight of the patient received significantly (*p*-value < 0.01) higher weight scores compared to body surface area and body mass index. Multiple comparisons of importance weights of items in the demographic characteristics of the patient category are shown in Additional file 4: Table S1.

#### Items related to prescribing medications

Consensus was also achieved on 16 items related to prompts with regards to the name of the medication, dose, frequency, route, formulation, units, duration, date, and who prescribed the medication. Prompts to specify the dose of the medication prescribed received significantly (*p*-value < 0.01) higher weight scores compared to specifying the formulation and other items. Multiple comparisons of importance weights of items in the prescribing medications category are shown in Additional file 4: Table S2.

#### Items related to checking prescriptions and alerts

Consensus was achieved on 16 items related to checking the suitability of the prescription in relation to the patient’s clinical and pathological conditions, discontinuing a medication, warnings of drug interactions, and justifying deviations from evidence-based prescribing. Ability to assess the suitability of the dose in relation to patient’s clinical and pathological conditions received significantly (*p*-value < 0.01) higher weight scores compared to warnings of potential contraindications. Multiple comparisons of importance weights of items in the checking prescriptions and alerts category are shown in Additional file 4: Table S3.

#### Items related to patient’s identity

Consensus was achieved on 14 items related to patient’s name, identification number, gender, date of birth, age, telephone number and address. Name of the patient received significantly (*p*-value < 0.05) higher weight scores compared to gender. Multiple comparisons of importance weights of items in the patient’s identity category are shown in Additional file 4: Table S4.

#### Items related to patient assessment

Consensus was achieved on 13 items related to prompts to enter clinical and pathological conditions of the patient, laboratory results, and patient history. Prompts to enter presenting symptoms of the patient received significantly (*p*-value < 0.05) higher weight scores compared to ability to enter and/or automatically import results of medical images ordered for the patient. Multiple comparisons of importance weights of items in the patient assessment category are shown in Additional file 4: Table S5.

#### Items related to quality of alerts

Consensus was achieved on 12 items related to providing evidence-based suggestions and alerts, ability to distinguish between serious and minor risks, and guiding the prescriber to best practices. Providing evidence-based suggestions and alerts received significantly (*p*-value < 0.01) higher weight scores compared to ability to distinguish serious and minor allergy. Multiple comparisons of importance weights of items in the quality of alerts category are shown in Additional file 4: Table S6.

#### Items related to admission and discharge of the patient

Consensus was achieved on 11 items related to details of the admission and discharge of the patient. Items related to the hospital’s admission number assigned to the patient at the time of admission received significantly (*p*-value < 0.05) higher weight scores compared to ability to name(s) of the ward(s) to which the patient was (were) admitted. Multiple comparisons of importance weights of items in the admission and discharge of the patient category are shown in Additional file 4: Table S7.

#### Items related to general features

Consensus was achieved on 9 items related to general features. The item related to a user friendly system received significantly (*p*-value < 0.05) higher weight scores compared to the item related to specifying why alerts are displayed. Multiple comparisons of importance weights of items in the general features category are shown in Additional file 4: Table S8.

#### Items related to diseases and making diagnosis

Consensus was achieved on 3 items related to diseases and making diagnosis. The item related to entering diagnosis received significantly (*p*-value < 0.05) higher weight scores compared to ability to access to offline, online, and searchable databases and references related to diseases and differential diagnosis. Multiple comparisons of importance weights of items in the diseases and making diagnosis category are shown in Additional file 4: Table S9.

#### Items on which consensus was not achieved

Following the third Delphi round, a total of 12 (9.8%) items remained equivocal (Table 4). Consensus was not achieved on items related to email address of parent(s)/guardian(s) of a pediatric patient, registration numbers of physicians, indicating why the medication was prescribed, ability to provide the prescriber with dosing recommendations, ability to check compatibility between medications and diluents, alerting prescribers of unlicensed medications for pediatric patients, verification of each medication administration by two persons, allowing search for all medications available in the country, collecting adverse reactions and prescribing errors, providing warnings to medications requiring monitoring and high alert medications.

**Table 4:** Features of EHRs with embedded CDSSs on which consensus was not achieved and remained as optional in the opinions of the panelists who participated in this study

		Round 02			Round 03			Source of the item
#	Item	M	IQR	%A	M	IQR	%A	
1	The email address of the parent(s)/guardian(s) of the pediatric patient	5	4	52	5	3	53	I
2	Registration number(s) of the physicians who prescribed the medications for the patients	4	5	45	4	4	43	L
3	Indication(s) for which the medication(s) was (were) prescribed	6	4	62	6	3	60	B
4	The system should be able to support the prescriber’s decisions by providing dosing recommendations	6	4	66	5	3	62	L
5	The system should be able to support the prescriber’s decisions by checking compatibility between medications and suggested diluents	6	3	62	6	4	67	I
6	The system should be able to support the prescriber’s decisions by providing a warning when the medication prescribed is not licensed for use in pediatric patients	6	4	62	6	3	60	L
7	Clear instructions that all medication administrations and checking should be verified by two persons	7	3	70	7	2	85	L
8	Allowing search and/or providing a mode of selection (for example a drop-down menu) for all medications licensed (available) in the country including their non-proprietary names and brand (branded-generic) names	5	4	68	6	3	71	B
9	Ability to collect adverse reactions attributed to medication use	4	4	41	5	3	46	L
10	Ability to provide warnings regarding medications that need monitoring	4	5	42	6	2	52	L
11	Ability to provide warnings (cautions) when high alert medications are prescribed	6	3	49	6	3	53	L
12	Ability to report prescribing errors	4	4	38	5	4	43	L

*%A* percentage of panelists who voted 7–9 on the item, *B* both (literature and interviews), *CDSSs* clinical decisions support systems, *EHRs* electronic health records, *I* interviews, *IQR* interquartile range, *L* literature, *M* median

## Discussion

In the present study, consensus was sought on a core list of important safety features to be considered when planning for, designing, developing, implementing, piloting, evaluating, maintaining, upgrading, and/or using EHRs with CDSSs. Currently, little guidance is available on what safety features are important to consider when planning for, designing, developing, implementing, piloting, evaluating, maintaining, upgrading, and/or using EHRs with CDSSs. This study presents for the first time a comprehensive consensus core list of 110 important safety features categorized into 9 categories that could be useful in guiding decision makers in hospitals, IT/programming industry, clinicians, and other healthcare provision team to design and improve EHRs and CDSSs. Keeping in mind that it can be difficult to consider all of these items, decision makers might need to select some items considering their relative importance weights.

This study was conducted in different stages using a mixed method (Fig. 1). The initial list of items that were collected after an extensive literature search and review followed by interviews with key contact experts in the field. The decision to conduct a thorough search instead of a systematic review was made after carefully considering the following issues: 1) objectives of the current study, 2) nature of the research question, 3) problem/population, intervention, comparison, outcome (PICO), 4) scope, and 5) number and nature of papers/materials to be included in the study [87]. Moreover, all panelists invited took part in the second Delphi round. This high response rate adds strength and validity to the design and findings of this study [22, 64–68, 74, 81]. The panel size was within the range of sizes used in previous studies involving developing concepts and achieving consensus on issues in healthcare [22, 41, 64–68, 72–81]. Despite the small size of the panel, both genders, different age groups, specialties/ranks/hierarchies, and type of employer were represented all represented (Table 1). Such diversity can add to the validity and suitability of using the consensus core items when planning for, designing, developing, implementing, piloting, evaluating, maintaining, upgrading, and/or using EHRs with CDSSs.

In general, the majority of the panelists agreed that EHRs with embedded CDSSs can improve patient’s safety, costs, record keeping, and workflow compared to paper-based handwritten patient records (Table 2). The panelists in this study, generally seemed to agree with findings reported in the literature as EHRs with embedded CDSSs improved the quality of healthcare delivery, increased time efficiency, improved adherence to guidelines, reduced medication errors and adverse medication events [5, 40]. However, some panelists were neutral or even disagreed with some statements and cited negative effects of EHRs with embedded CDSSs in relation to patient’s safety, costs, record keeping, and workflow in agreement with those reported in the literature [29, 44, 52].

Gold standards in guiding the design and development of systems EHRs with embedded CDSSs do not exist. When gold standards are absent, stakeholders are left wondering what items are important to consider when designing such systems. In this case, consensus achieving approaches might be useful in reducing bias, increase transparency, and adding strength to judgmental approaches [88]. It has been argued that professionals tend to adhere to guidelines when they agree with them compared to guidelines they do not agree with. Therefore in this case, it is believed that decision makers in both health provision team and IT/programming are expected to consider such consensus items when planning for, designing, developing, implementing, piloting, evaluating, maintaining, upgrading, and/or using EHRs with CDSSs.

In this study, consensus was achieved that the system should be able to record and keep admission and discharge information of the patient (Table 3). Such information might be used to answer why, when, where, and how questions, like when the patient was admitted to the hospital, why the patient was admitted, where the patient was admitted (ward and bed number), who admitted and who discharged the patient. Such information can be indispensable in tracking the patient’s history and retrieving the patient’s information whenever needed. Consensus was also achieved on items related to the patient’s identify and body characteristics. Body weight and surface area might be used by the prescribers to calculate the dose for example, especially in pediatric patients [89, 90]. The panelists also agreed that features need to include prompts and abilities to make entries related to patient assessment like presenting symptoms, vital signs, laboratory and imaging reports, co-morbidities, prescription and non-prescription medications, allergies, and adverse drug reactions. Such features might improve diagnostic accuracy [35, 42, 48, 51], effectiveness of medication review and patient care [36–38, 46], improve screening for allergies to medications [34], reduced adverse drug reactions [33], drug-drug, and drug-food interactions [4, 49, 50]. The panelists also agreed that the system should provide prompts to specifying doses of the medications prescribed, frequencies, routes of administration, dosage forms, dosing units, alternative medications, and duration of therapy. Such features were shown to reduce prescribing errors and administration errors [39, 43, 45]. Previous studies have shown that medication errors increased the length of hospital stays, costs of therapy and often resulted in death of the patient [91]. Consensus was also achieved on items related to alerts provided by the system after checking prescriptions. Previous studies have shown usefulness of EHRs with embedded CDSSs in assessing appropriateness and safety of doses in relation to renal function [32], drug allergies, contraindications, adverse drug reactions, drug-drug, and drug-food interactions [19, 33, 50]. It is noteworthy mentioning that in order to improve diagnostic accuracy and patient care, systems should provide the healthcare provider with up-to-date information and evidence [21]. In this study, consensus was achieved on items related to the quality of alerts and warnings provided by the system. The panelists agreed on items related to the ease of using the system (Table 3).

In the present study, consensus was not achieved to include items like the email address of the parent(s)/guardian(s) of the pediatric patient, registration number of the prescriber, indication for which the medication was prescribed, dosing recommendations, compatibility with diluents, unlicensed use of a medication, all medications available in the country, collecting adverse drug reactions, using high alert medications, and reporting medication errors. The decision to either consider these items or not is left to the decision makers when planning for, designing, developing, implementing, piloting, evaluating, maintaining, upgrading, and/or using EHRs with CDSSs. The decision to whether consider these items or not might be shaped by the individual needs of healthcare providers and decision makers in hospitals.

This study is not without limitations. First, a systematic review of the literature was not conducted in this study. Compared to qualitative reviews, systematic reviews are more robust and allow reproducibility of results. Second, data extraction was conducted by one investigator. Although another researcher verified the results independently, the risk of bias could have been reduced if two or more researchers independently conducted the data extraction step. One investigator summarized, extracted, and grouped the qualitative data. Items were directly entered into the data collection form as interpreted from the recordings. Although the participants had the opportunity to read and correct the summaries, risk of bias could have been reduced if two or more researchers independently summarized, extracted, and grouped the qualitative data or if the exact words of the interviewees were transcribed. Third, patients were not included as panelists in this study. Including patients in the panel should have allowed to explore patient’s views with regards to EHRs with CDSSs. In modern healthcare systems, patients are increasingly involved in designing their care plans. Fourth, the panel of experts recruited for this study was relatively small. However, there is no consensus on the number of panelists that should be included in a Delphi study [78]. Previous studies in healthcare used panel sizes in the range of 10–50 [64–68, 74]. In this study, care was exerted and a diverse panel was composed to include physicians, nurses, hospital pharmacists, and IT/programming specialists. Fifth, the panelists were identified by key contacts in the field and were recruited using a purposive sampling technique. Such sampling technique has long been criticized as biased [64–68, 74]. However, prior knowledge of the subject being investigated is a pre-requisite for potential participants to be selected into a panel. Finally, this study could have been conducted in a conference or large scientific meeting in which experts with interest in EHRs with embedded CDSSs would gather.

## Conclusions

In this study, merits, features, and desiderata to be considered when planning for, designing, developing, implementing, piloting, evaluating, maintaining, upgrading, and/or using EHRs with CDSSs were explored. Consensus was achieved on items related to safety features to be considered when planning for, designing, developing, implementing, piloting, evaluating, maintaining, upgrading, and/or using EHRs with CDSSs. Considering items on which consensus was achieved might promote congruence and safety while planning for, designing, developing, implementing, piloting, evaluating, maintaining, upgrading, and/or using EHRs with CDSSs. Further studies are still needed to determine if these recommendations can improve patient safety and outcomes in Palestinian hospitals.

## Supplementary information

**Additional file 1.** Adherence to PRISMA guidelines.

**Additional file 2.** Adherence to COnsolidated criteria for REporting Qualitative research (COREQ) Checklist.

**Additional file 3.** Adherence to Conducting and REporting of DElphi Studies (CREDES) guidelines.

**Additional file 4: Tables S1-S9.** show multiple comparisons of importance weights items in each category.

## Data Availability

Data related to this study are either presented in the results section or in the additional files. Raw data can be obtained from the corresponding author on reasonable request.

## Abbreviations

%A: Percentage of panelists who voted 7–9 on the item
ANOVA: Analysis of variance
B: Both (literature and interviews)
BSc: Bachelor of Science
CDSSs: Clinical decision support systems
COREQ: Consolidated criteria for reporting qualitative research
CREDES: Conducting and reporting of Delphi studies
EHRs: Electronic health records
HIS: Health information system
I: Interviews
IQR: Interquartile range
IRB: Institutional Review Board
IT: Information technology
L: Literature
M: Median
MD: Doctor of Medicine
MSc: Master of Science
NA: Not applicable
Pharm.D: Doctor of Pharmacy
PhD: Doctor of Philosophy
PICO: Problem/population, intervention, comparison, outcome
Q1: 1st quartile
Q2: 2nd quartile
Q3: 3rd quartile
SD: Standard deviation
UNRWA: United Nations Relief and Works Agency for Palestine Refugees
