# Supplementary material for: Merits, features, and desiderata to be considered when developing electronic health records with embedded clinical decision support systems in Palestinian hospitals: a consensus study
Source: BMC Med Inform Decis Mak. 2019 Nov 8;19:216. doi: 10.1186/s12911-019-0928-3 (PMC6842153; doi:10.1186/s12911-019-0928-3)
Supplement: Supplementary file 1 — Additional file 1. Adherence to PRISMA guidelines. [file 12911_2019_928_MOESM1_ESM.docx]

**Additional file 1:** Adherence to PRISMA guidelines [[1](#_ENREF_1)]

| **#** | **Section/topic** | **Checklist item** | **What was applied** | **Page/Line # in the manuscript** |
| --- | --- | --- | --- | --- |
|  | **TITLE** | |  |  |
| 1 | Title | Identify the report as a systematic review, meta-analysis, or both. | As this was not a systematic review, this item was not applied | - |
|  | **ABSTRACT** | |  |  |
| 2 | Structured summary | Provide a structured summary including, as applicable: background; objectives; data sources; study eligibility criteria, participants, and interventions; study appraisal and synthesis methods; results; limitations; conclusions and implications of key findings; systematic review registration number. | The abstract mentioned that a thorough search of the literature was performed. | Abstract, methods section |
|  | **INTRODUCTION** | |  |  |
| 3 | Rationale | Describe the rationale for the review in the context of what is already known. | The rationale behind conducting the thorough literature search and review was described in the methods section. | Page 5 Lines 30-32. |
| 4 | Objectives | Provide an explicit statement of questions being addressed with reference to participants, interventions, comparisons, outcomes, and study design (PICOS). | The objective of the literature search and review was provided. | Page 5 Lines 32-37. |
|  | **METHODS** | |  |  |
| 5 | Protocol and registration | Indicate if a review protocol exists, if and where it can be accessed (e.g., Web address), and, if available, provide registration information including registration number. | N/A | - |
| 6 | Eligibility criteria | Specify study characteristics (e.g., PICOS, length of follow-up) and report characteristics (e.g., years considered, language, publication status) used as criteria for eligibility, giving rationale. | Eligibility criteria were described in the methods section. | Page 6 Lines 8-14. |
| 7 | Information sources | Describe all information sources (e.g., databases with dates of coverage, contact with study authors to identify additional studies) in the search and date last searched. | Databases, search engines were provided in the in the methods section. , | Page 5 Lines 38-40. Page 6 Lines 5-6. |
| 8 | Search | Present full electronic search strategy for at least one database, including any limits used, such that it could be repeated. | The search strategy, key terms, and dates were described in the methods section. | Page 5 Lines 39-40. Page 6 Lines 1-14. |
| 9 | Study selection | State the process for selecting studies (i.e., screening, eligibility, included in systematic review, and, if applicable, included in the meta-analysis). | Screening, eligibility for inclusion were described in the methods section. | Page 6 Lines 6-11 and Lines 15-19. |
| 10 | Data collection process | Describe method of data extraction from reports (e.g., piloted forms, independently, in duplicate) and any processes for obtaining and confirming data from investigators. | Data collection was described under Extraction of items section. | Page 6 Lines 20-29. |
| 11 | Data items | List and define all variables for which data were sought (e.g., PICOS, funding sources) and any assumptions and simplifications made. | Described in the methods section. | Page 5 Lines 30-32, Page 6 Lines 11-13. |
| 12 | Risk of bias in individual studies | Describe methods used for assessing risk of bias of individual studies (including specification of whether this was done at the study or outcome level), and how this information is to be used in any data synthesis. | N/A | - |
| 13 | Summary measures | State the principal summary measures (e.g., risk ratio, difference in means). | N/A | - |
| 14 | Synthesis of results | Describe the methods of handling data and combining results of studies, if done, including measures of consistency (e.g., I^2^) for each meta-analysis. | Described under Extraction of items section | Page 6 Lines 20-29. |
| 15 | Risk of bias across studies | Specify any assessment of risk of bias that may affect the cumulative evidence (e.g., publication bias, selective reporting within studies). | N/A | - |
| 16 | Additional analyses | Describe methods of additional analyses (e.g., sensitivity or subgroup analyses, meta-regression), if done, indicating which were pre-specified. | N/A  (additional interviews were conducted and described) | Page 6 Lines 30-44 and Page 7 Lines 1-18. |
|  | **RESULTS** | |  |  |
| 17 | Study selection | Give numbers of studies screened, assessed for eligibility, and included in the review, with reasons for exclusions at each stage, ideally with a flow diagram. | Described in the results section. As this was not a systematic review, the PRISMA diagram was not included. | Page 11 Lines 3-4. |
| 18 | Study characteristics | For each study, present characteristics for which data were extracted (e.g., study size, PICOS, follow-up period) and provide the citations. | N/A | - |
| 19 | Risk of bias within studies | Present data on risk of bias of each study and, if available, any outcome level assessment (see item 12). | N/A | - |
| 20 | Results of individual studies | For all outcomes considered (benefits or harms), present, for each study: (a) simple summary data for each intervention group (b) effect estimates and confidence intervals, ideally with a forest plot. | N/A | - |
| 21 | Synthesis of results | Present results of each meta-analysis done, including confidence intervals and measures of consistency. | N/A | - |
| 22 | Risk of bias across studies | Present results of any assessment of risk of bias across studies (see Item 15). | N/A | - |
| 23 | Additional analysis | Give results of additional analyses, if done (e.g., sensitivity or subgroup analyses, meta-regression [see Item 16]). | Results of the interviews were presented under the results section. | Page 11 Lines 7-14. |
|  | **DISCUSSION** | |  |  |
| 24 | Summary of evidence | Summarize the main findings including the strength of evidence for each main outcome; consider their relevance to key groups (e.g., healthcare providers, users, and policy makers). | N/A | - |
| 25 | Limitations | Discuss limitations at study and outcome level (e.g., risk of bias), and at review-level (e.g., incomplete retrieval of identified research, reporting bias). | Limitations with risk of bias are discussed in the discussion section. | Page 16, Lines 26-45. |
| 26 | Conclusions | Provide a general interpretation of the results in the context of other evidence, and implications for future research. | N/A | - |
|  | **FUNDING** | |  |  |
| 27 | Funding | Describe sources of funding for the systematic review and other support (e.g., supply of data); role of funders for the systematic review. | N/A | - |

N/A: not applicable

**Reference:**

1. Moher D, Liberati A, Tetzlaff J, Altman DG, Group P. Preferred reporting items for systematic reviews and meta-analyses: the PRISMA statement. J Clin Epidemiol. 2009. https://doi.org/10.1016/j.jclinepi.2009.06.005.
