# Supplementary material for: Merits, features, and desiderata to be considered when developing electronic health records with embedded clinical decision support systems in Palestinian hospitals: a consensus study
Source: BMC Med Inform Decis Mak. 2019 Nov 8;19:216. doi: 10.1186/s12911-019-0928-3 (PMC6842153; doi:10.1186/s12911-019-0928-3)
Supplement: Supplementary file 2 — Additional file 2. Adherence to COnsolidated criteria for REporting Qualitative research (COREQ) Checklist. [file 12911_2019_928_MOESM2_ESM.docx]

**Additional file 2:** Adherence to COnsolidated criteria for REporting Qualitative research (COREQ) Checklist [[1](#_ENREF_1)]

| **#** | **Topic** | **Guide Questions/Description** | **Page/Line # in the manuscript** |
| --- | --- | --- | --- |
|  | **Domain 1: Research team and reﬂexivity** |  |  |
|  | *Personal characteristics* |  |  |
| 1 | Interviewer/facilitator | Which author/s conducted the interview or focus group? | Page 6 Line 44 |
| 2 | Credentials | What were the researcher’s credentials? E.g. PhD, MD | Page 6 Line 44 |
| 3 | Occupation | What was their occupation at the time of the study? | Page 7 Lines 1-2 |
| 4 | Gender | Was the researcher male or female? | Page 7 Line 1 |
| 5 | Experience and training | What experience or training did the researcher have? | Page 7 Lines 1-2 |
|  | *Relationship with participants* |  |  |
| 6 | Relationship established | Was a relationship established prior to study commencement? | Page 6 Lines 35-40. |
| 7 | Participant knowledge of the interviewer | What did the participants know about the researcher? e.g. personal goals, reasons for doing the research | Page 6 Lines 40-43. |
| 8 | Interviewer characteristics | What characteristics were reported about the inter viewer/facilitator? e.g. Bias, assumptions, reasons and interests in the research topic | Page 6 Lines 40-43. |
|  | **Domain 2: Study design** |  |  |
|  | *Theoretical framework* |  |  |
| 9 | Methodological orientation and Theory | What methodological orientation was stated to underpin the study? e.g. grounded theory, discourse analysis, ethnography, phenomenology, content analysis | Page 7 Lines 13-14. |
|  | *Participant selection* |  |  |
| 10 | Sampling | How were participants selected? e.g. purposive, convenience, consecutive, snowball | Page 6 Lines 35-36. |
| 11 | Method of approach | How were participants approached? e.g. face-to-face, telephone, mail, email | Page 7 Lines 5-6. |
| 12 | Sample size | How many participants were in the study? | Page 6 Lines 36. |
| 13 | Non-participation | How many people refused to participate or dropped out? Reasons? | Page 11 Lines 7-9. |
|  | *Setting* |  |  |
| 14 | Setting of data collection | Where was the data collected? e.g. home, clinic, workplace | Page 7 Lines 5-6. |
| 15 | Presence of non-participants | Was anyone else present besides the participants and researchers? | Page 7 Lines 5-6. |
| 16 | Description of sample | What are the important characteristics of the sample? e.g. demographic data, date | Page 11 Lines 7-9 and Table 1. |
|  | *Data collection* | | |
| 17 | Interview guide | Were questions, prompts, guides provided by the authors? Was it pilot tested? | Page 7 Lines 5-13. |
| 18 | Repeat interviews | Were repeat interviews carried out? If yes, how many? | Page 7 Lines 11-12. |
| 19 | Audio/visual recording | Did the research use audio or visual recording to collect the data? | Page 7 Lines 11-12. |
| 20 | Field notes | Were ﬁeld notes made during and/or after the interview or focus group? | Page 7 Lines 12-13. |
| 21 | Duration | What was the duration of the inter views or focus group? | Page 11 Lines 13-14. |
| 22 | Data saturation | Was data saturation discussed? | Page 7 Lines 14. |
| 23 | Transcripts returned | Were transcripts returned to participants for comment and/or correction? | Page 7 Lines 14-15. |
|  | **Domain 3: analysis and ﬁndings** |  |  |
|  | *Data analysis* | | |
| 24 | Number of data coders | How many data coders coded the data? | Page 9 Lines 1-2. |
| 25 | Description of the coding tree | Did authors provide a description of the coding tree? | N/A |
| 26 | Derivation of themes | Were themes identiﬁed in advance or derived from the data? | Page 7 Lines 14-15. |
| 27 | Software | What software, if applicable, was used to manage the data? | Page 9 Lines 2. |
| 28 | Participant checking | Did participants provide feedback on the ﬁndings? | Page 7 Lines 15-16. |
|  | *Reporting* |  |  |
| 29 | Quotations presented | Were participant quotations presented to illustrate the themes/ﬁndings? Was each quotation identiﬁed? e.g. participant number | Table 2. |
| 30 | Data and ﬁndings consistent | Was there consistency between the data presented and the ﬁndings? | N/A |
| 31 | Clarity of major themes | Were major themes clearly presented in the ﬁndings? | Tables 2-4. |
| 32 | Clarity of minor themes | Is there a description of diverse cases or discussion of minor themes? | Tables 2-4. |

**Reference:**

1. Tong A, Sainsbury P, Craig J. Consolidated criteria for reporting qualitative research (COREQ): a 32-item checklist for interviews and focus groups. Int J Qual Health Care. 2007. https://doi.org/10.1093/intqhc/mzm042.
