# Supplementary material for: Merits, features, and desiderata to be considered when developing electronic health records with embedded clinical decision support systems in Palestinian hospitals: a consensus study
Source: BMC Med Inform Decis Mak. 2019 Nov 8;19:216. doi: 10.1186/s12911-019-0928-3 (PMC6842153; doi:10.1186/s12911-019-0928-3)
Supplement: Supplementary file 3 — Additional file 3. Adherence to Conducting and REporting of DElphi Studies (CREDES) guidelines. [file 12911_2019_928_MOESM3_ESM.docx]

**Additional file 3:** Adherence to Conducting and REporting of DElphi Studies (CREDES) guidelines [[1](#_ENREF_1)]

| **#** | **Category** | **Page/line # in the manuscript** |
| --- | --- | --- |
|  | **Rationale for the choice of the Delphi technique** |  |
| 1 | Justification/rationale for Delphi | Introduction: Page 3, Lines 43-44, Page 4 Lines 2-4. |
| 2 | Purpose well defined | Introduction: Page 3, Lines 5-11. |
|  | **Planning and design** |  |
| 1 | Planning and process | Methods: Page 5, lines 29-40, Page 6, Lines 1-29, 30-44, Page 7 Lines 1-20. |
| 2 | Selection of experts clearly justified | Methods: Page 7, Lines 2-6, Page 8 Lines 6-35. |
|  | **Study conduct** |  |
| 1 | Clear description of methods | Methods: Page 5-9. |
| 2 | Flow chart | Figure 1. |
| 3 | Informational input | Methods: Page 5, section: Literature search, Page 6 section: The first Delphi round: interviews with key contact experts. |
| 4 | Clear definition of consensus | Methods: Page 9, section: Definition of consensus. |
| 5 | Prevention of bias | Methods: Page 7, Lines 11-17. |
| 6 | Pilot test of instruments | Methods: Page 8, Lines 1-5. |
| 7 | Interpretation and processing of results | Results: Page 11, Lines 2-23. |
| 8 | Validity | Discussion: Page 15, Lines 11-20. |
|  | **Reporting** |  |
| 1 | Transparent reporting of results | Results: Page 11, Lines 2-23, Tables 1-4 and Additional Tables A1-A9 and additional file 3. |
| 2 | Data analysis clearly justified and reported | Results: Page 11, Lines 2-23, Tables 1-4 and Additional Tables A1-A9 and additional file 3. |
| 3 | Expert panel (Member of organization, recognized authority, relevant clinical academic expertise, profession/stakeholder) | Methods: Page 8, Lines 6-35 and Table 1. |
| 4 | Information of rounds | Results: Page 11, Lines 2-23, Tables 1-4 and Additional Tables A1-A9 and additional file 1. |
| 5 | Discussion of limitations | Discussion: Page 16, Lines 26-44. |
| 6 | Adequacy of conclusions | Discussion: Page 17, Lines 2-9. |

**Reference:**

1. Junger S, Payne SA, Brine J, Radbruch L, Brearley SG. Guidance on Conducting and REporting DElphi Studies (CREDES) in palliative care: Recommendations based on a methodological systematic review. Palliat Med. 2017. https://doi.org/10.1177/0269216317690685.
