# Supplementary material for: Merits, features, and desiderata to be considered when developing electronic health records with embedded clinical decision support systems in Palestinian hospitals: a consensus study
Source: BMC Med Inform Decis Mak. 2019 Nov 8;19:216. doi: 10.1186/s12911-019-0928-3 (PMC6842153; doi:10.1186/s12911-019-0928-3)
Supplement: Supplementary file 4 — Additional file 4: Tables S1-S9. show multiple comparisons of importance weights items in each category. [file 12911_2019_928_MOESM4_ESM.docx]

**Additional file 4:** Multiple comparisons of weights given by the panelists in the Analytic Hierarchy Process on items in each of the 9 categories

**Additional Table S1:** Multiple comparisons of weights given by the panelists in the Analytic Hierarchy Process to each item in the demographic characteristics of the patient category

| **Item #** | **1** | **2** | **3** | **4** | **5** | **6** | **7** | **8** | **9** | **10** | **11** | **12** | **13** | **14** | **15** | **16** |
| --- | --- | --- | --- | --- | --- | --- | --- | --- | --- | --- | --- | --- | --- | --- | --- | --- |
| **1** | - | ns | ns | ns | ns | ns | ** | ** | *** | **** | **** | **** | **** | **** | **** | **** |
| **2** | ns | - | ns | ns | ns | ns | ** | ** | *** | **** | **** | **** | **** | **** | **** | **** |
| **3** | ns | ns | - | ns | ns | ns | ns | ns | ns | ** | ** | *** | **** | **** | **** | **** |
| **4** | ns | ns | ns | - | ns | ns | ns | ns | ns | ** | ** | *** | **** | **** | **** | **** |
| **5** | ns | ns | ns | ns | - | ns | ns | ns | ns | ns | ns | ** | *** | *** | *** | **** |
| **6** | ns | ns | ns | ns | ns | - | ns | ns | ns | ns | ns | ** | *** | *** | *** | **** |
| **7** | ** | ** | ns | ns | ns | ns | - | ns | ns | ns | ns | ns | ** | ** | ** | *** |
| **8** | ** | ** | ns | ns | ns | ns | ns | - | ns | ns | ns | ns | ** | ** | ** | *** |
| **9** | *** | *** | ns | ns | ns | ns | ns | ns | - | ns | ns | ns | ns | ns | ns | ** |
| **10** | **** | **** | ** | ** | ns | ns | ns | ns | ns | - | ns | ns | ns | ns | ns | ns |
| **11** | **** | **** | ** | ** | ns | ns | ns | ns | ns | ns | - | ns | ns | ns | ns | ns |
| **12** | **** | **** | *** | *** | ** | ** | ns | ns | ns | ns | ns | - | ns | ns | ns | ns |
| **13** | **** | **** | **** | **** | *** | *** | ** | ** | ns | ns | ns | ns | - | ns | ns | ns |
| **14** | **** | **** | **** | **** | *** | *** | ** | ** | ns | ns | ns | ns | ns | - | ns | ns |
| **15** | **** | **** | **** | **** | *** | *** | ** | ** | ns | ns | ns | ns | ns | ns | - | ns |
| **16** | **** | **** | **** | **** | **** | **** | *** | *** | ** | ns | ns | ns | ns | ns | ns | - |

1: The body weight of the patient, 2: The measure units of weight (gm, kg, pounds), 3: The working weight of the patient that was used for dose calculations (for example, 5 kg instead of 5.1 kg), 4: Date on which the weight of the patient was measured, 5: The height of the patient, 6: The measure units for height (cm, m, in), 7: The date on which the height of the patient was measured, 8: The body surface area of the pediatric patient, 9: The measure units of the body surface area, 10: The date on which the body surface area was measured, 11: The body mass index of the patient, 12: The date on which the body mass index of the patient was measured, 13: Time on which the weight of the patient was measured, 14: The time on which the height of the patient was measured, 15: The time on which the body surface area was measured, 16: The time on which the body mass index of the patient was measured

Comparisons were performed using ANOVA with Bonferroni's multiple comparisons, NS: not significant, *: *p*-value < 0.05, **: *p*-value < 0.01, ***: *p*-value < 0.001, ****: *p*-value < 0.0001.

**Additional Table S2:** Multiple comparisons of weights given by the panelists in the Analytic Hierarchy Process to each item in the prescribing medications category

| **Item #** | **1** | **2** | **3** | **4** | **5** | **6** | **7** | **8** | **9** | **10** | **11** | **12** | **13** | **14** | **15** | **16** |
| --- | --- | --- | --- | --- | --- | --- | --- | --- | --- | --- | --- | --- | --- | --- | --- | --- |
| **1** | - | ns | ns | ** | *** | **** | **** | **** | **** | **** | **** | **** | **** | **** | **** | **** |
| **2** | ns | - | ns | ns | ns | ns | ns | * | ** | *** | *** | **** | **** | **** | **** | **** |
| **3** | ns | ns | - | ns | ns | ns | ns | ns | ns | ** | ** | *** | **** | **** | **** | **** |
| **4** | ** | ns | ns | - | ns | ns | ns | ns | ns | ns | * | * | ** | **** | **** | **** |
| **5** | *** | ns | ns | ns | - | ns | ns | ns | ns | ns | ns | ns | * | ** | *** | **** |
| **6** | **** | ns | ns | ns | ns | - | ns | ns | ns | ns | ns | ns | ns | * | ** | **** |
| **7** | **** | ns | ns | ns | ns | ns | - | ns | ns | ns | ns | ns | ns | ns | * | ** |
| **8** | **** | * | ns | ns | ns | ns | ns | - | ns | ns | ns | ns | ns | ns | ns | ** |
| **9** | **** | ** | ns | ns | ns | ns | ns | ns | - | ns | ns | ns | ns | ns | ns | * |
| **10** | **** | *** | ** | ns | ns | ns | ns | ns | ns | - | ns | ns | ns | ns | ns | ns |
| **11** | **** | *** | ** | * | ns | ns | ns | ns | ns | ns | - | ns | ns | ns | ns | ns |
| **12** | **** | **** | *** | * | ns | ns | ns | ns | ns | ns | ns | - | ns | ns | ns | ns |
| **13** | **** | **** | **** | ** | * | ns | ns | ns | ns | ns | ns | ns | - | ns | ns | ns |
| **14** | **** | **** | **** | **** | ** | * | ns | ns | ns | ns | ns | ns | ns | - | ns | ns |
| **15** | **** | **** | **** | **** | *** | ** | * | ns | ns | ns | ns | ns | ns | ns | - | ns |
| **16** | **** | **** | **** | **** | **** | **** | ** | ** | * | ns | ns | ns | ns | ns | ns | - |

1: Prompting a mode of selection for specifying the dose (for example mg, µg, mL, …etc.) of the medication prescribed, 2: Prompting a mode of selection for specifying the frequency (number of times) the medication needs to be administered (for example, once daily, twice daily, three times daily, ..etc.), 3: Prompting a mode of selection for specifying the route by which the medication would be administered (for example, oral, intravenous, intramuscular, …etc.), 4: Prompting a mode of selection for specifying the dosage form "formulation" (for example, tablet, capsule, syrup, ..etc.) of the medication prescribed, 5: Prompting a mode of selection for specifying the number of dosing units to be administered each time (for example, one tablet, two tablets, …etc.), 6: Allowing search and/or providing a mode of selection (for example a drop-down menu) for all medications available on the hospital's formulary including their non-proprietary names and brand (branded-generic) names, 7: Prompting a mode of selection for specifying the date on which the medication was prescribed, 8: Prompting a mode of selection for specifying the duration for which the medication administration should be continued, 9: Prompting a mode of selection for specifying the date on which the medication administration should be started, 10: Prompting a mode of selection for specifying the times at which the medication doses should be administered (for example, at 8:00 am, 2:00 pm, …etc.), 11: Prompting a mode of selection for specifying the date on which the medication administration should be discontinued, 12: Prompting a mode of selection for specifying the time on which the medication administration should be discontinued, 13: Prompting a mode of selection for specifying the medication administration in relation to meals, 14: Prompting a mode of selection for specifying the maximal number of doses to be administered in 24 hours for medication prescribed as "when needed" (PRN), 15: Prompting a mode of selection for specifying the name of the physician who prescribed the medication, 16: Ability to suggest other suitable substitutes (other medications from the same pharmacological class)

Comparisons were performed using ANOVA with Bonferroni's multiple comparisons, NS: not significant, *: *p*-value < 0.05, **: *p*-value < 0.01, ***: *p*-value < 0.001, ****: *p*-value < 0.0001.

**Additional Table S3:** Multiple comparisons of weights given by the panelists in the Analytic Hierarchy Process to each item in the checking prescriptions and alerts category

| **Item #** | **1** | **2** | **3** | **4** | **5** | **6** | **7** | **8** | **9** | **10** | **11** | **12** | **13** | **14** | **15** | **16** |
| --- | --- | --- | --- | --- | --- | --- | --- | --- | --- | --- | --- | --- | --- | --- | --- | --- |
| **1** | - | ns | ns | ** | *** | **** | **** | **** | **** | **** | **** | **** | **** | **** | **** | **** |
| **2** | ns | - | ns | ns | * | *** | **** | **** | **** | **** | **** | **** | **** | **** | **** | **** |
| **3** | ns | ns | - | ns | ns | ns | * | *** | **** | **** | **** | **** | **** | **** | **** | **** |
| **4** | ** | ns | ns | - | ns | ns | ns | * | **** | **** | **** | **** | **** | **** | **** | **** |
| **5** | *** | * | ns | ns | - | ns | ns | ns | *** | **** | **** | **** | **** | **** | **** | **** |
| **6** | **** | *** | ns | ns | ns | - | ns | ns | * | *** | **** | **** | **** | **** | **** | **** |
| **7** | **** | **** | * | ns | ns | ns | - | ns | ns | * | *** | **** | **** | **** | **** | **** |
| **8** | **** | **** | *** | * | ns | ns | ns | - | ns | ns | ** | ** | *** | *** | **** | **** |
| **9** | **** | **** | **** | **** | *** | * | ns | ns | - | ns | ns | ns | ns | * | ** | ** |
| **10** | **** | **** | **** | **** | **** | *** | * | ns | ns | - | ns | ns | ns | ns | ns | ns |
| **11** | **** | **** | **** | **** | **** | **** | *** | ** | ns | ns | - | ns | ns | ns | ns | ns |
| **12** | **** | **** | **** | **** | **** | **** | **** | ** | ns | ns | ns | - | ns | ns | ns | ns |
| **13** | **** | **** | **** | **** | **** | **** | **** | *** | ns | ns | ns | ns | - | ns | ns | ns |
| **14** | **** | **** | **** | **** | **** | **** | **** | *** | * | ns | ns | ns | ns | - | ns | ns |
| **15** | **** | **** | **** | **** | **** | **** | **** | **** | ** | ns | ns | ns | ns | ns | - | ns |
| **16** | **** | **** | **** | **** | **** | **** | **** | **** | ** | ns | ns | ns | ns | ns | ns | - |

1: Ability to assess suitability of the dose in view of the patient's conditions like renal and/or hepatic functions, 2: Clear instructions to guide prescribers on the procedures to follow when a medication order to be discontinued or changed, 3: Ability to check for and provide warnings on potential drug-drug interactions, 4: Ability to check for and provide warnings on potentially contraindicated medications for the patient, 5: Ability to check for and provide warnings on potential drug-food interactions, 6: Ability to check for and provide warnings on potential drug-herb interactions, 7: Ability to provide warnings regarding any potential medication adverse reactions in view of the patient's conditions, 8: Ability to recommend evidence-based dose suitable for the patient, 9: Ability to check for and provide warning when another medication from the same pharmacological class (duplication) is prescribed, 10: Ability to provide prompts on special precautions or procedures to administer the prescribed medication (if any), 11: Ability to alert the prescriber if the dosage form(s) prescribed was (were) of slow or modified release, 12: Ability to enter reason(s) (justification) why another medication from the same pharmacological class (duplication) is prescribed, 13: Ability to add reasons (justification) for not changing the medication or dose in the event of an adverse medication reaction, 14: Ability to enter reason(s) (justification) why the dose was different from the evidence-based recommended one, 15: Ability to enter reason(s) (justification) why the dosing frequency was different from the evidence-based recommended one, 16: Ability to enter reason(s) (justification) why the duration of medication administration was different from the evidence-based recommended one

Comparisons were performed using ANOVA with Bonferroni's multiple comparisons, NS: not significant, *: *p*-value < 0.05, **: *p*-value < 0.01, ***: *p*-value < 0.001, ****: *p*-value < 0.0001.

**Additional Table S4:** Multiple comparisons of weights given by the panelists in Analytic Hierarchy Process to each item in the patient's identity category

| **Item #** | **1** | **2** | **3** | **4** | **5** | **6** | **7** | **8** | **9** | **10** | **11** | **12** | **13** | **14** |
| --- | --- | --- | --- | --- | --- | --- | --- | --- | --- | --- | --- | --- | --- | --- |
| **1** | - | ns | ns | ns | ns | * | * | *** | *** | **** | **** | **** | **** | **** |
| **2** | ns | - | ns | ns | ns | ns | ns | * | * | *** | **** | **** | **** | **** |
| **3** | ns | ns | - | ns | ns | ns | ns | * | * | *** | **** | **** | **** | **** |
| **4** | ns | ns | ns | - | ns | ns | ns | ns | ns | ns | * | *** | **** | **** |
| **5** | ns | ns | ns | ns | - | ns | ns | ns | ns | ns | * | *** | **** | **** |
| **6** | * | ns | ns | ns | ns | - | ns | ns | ns | ns | ns | * | **** | **** |
| **7** | * | ns | ns | ns | ns | ns | - | ns | ns | ns | ns | * | **** | **** |
| **8** | *** | * | * | ns | ns | ns | ns | - | ns | ns | ns | ns | *** | **** |
| **9** | *** | * | * | ns | ns | ns | ns | ns | - | ns | ns | ns | *** | **** |
| **10** | **** | *** | *** | ns | ns | ns | ns | ns | ns | - | ns | ns | * | ** |
| **11** | **** | **** | **** | * | * | ns | ns | ns | ns | ns | - | ns | ns | ns |
| **12** | **** | **** | **** | *** | *** | * | * | ns | ns | ns | ns | - | ns | ns |
| **13** | **** | **** | **** | **** | **** | **** | **** | *** | *** | * | ns | ns | - | ns |
| **14** | **** | **** | **** | **** | **** | **** | **** | **** | **** | ** | ns | ns | ns | - |

1: The first name of the patient, 2: The father's name of the patient, 3: The grandfather's name of the patient, 4: The family name (surname) of the patient, 5: The unique national identification number of the patient, 6: The gender of the patient, 7: The date of birth of the patient, 8: The age of the patient, 9: The measure units of age (years, months, or days), 10: The gestational age of the pediatric patient (for neonates), 11: The corrected gestational age of the pediatric patient (if the neonate was a preterm), 12: The date on which the age of the patient was calculated, 13: The telephone number of the patient/their parent(s)/guardian(s) in case of a pediatric patient, 14: The home address of the patient

Comparisons were performed using ANOVA with Bonferroni's multiple comparisons, NS: not significant, *: *p*-value < 0.05, **: *p*-value < 0.01, ***: *p*-value < 0.001, ****: *p*-value < 0.0001.

**Additional Table S5:** Multiple comparisons of weights given by the panelists in the Analytic Hierarchy Process to each item in the patient assessment category

| **Item #** | **1** | **2** | **3** | **4** | **5** | **6** | **7** | **8** | **9** | **10** | **11** | **12** | **13** |
| --- | --- | --- | --- | --- | --- | --- | --- | --- | --- | --- | --- | --- | --- |
| **1** | - | ns | ns | * | ** | *** | **** | **** | **** | **** | **** | **** | **** |
| **2** | ns | - | ns | ns | ns | * | ** | *** | **** | **** | **** | **** | **** |
| **3** | ns | ns | - | ns | ns | ns | ns | * | ** | *** | **** | **** | **** |
| **4** | * | ns | ns | - | ns | ns | ns | ns | ns | * | *** | **** | **** |
| **5** | ** | ns | ns | ns | - | ns | ns | ns | ns | ns | *** | *** | **** |
| **6** | *** | * | ns | ns | ns | - | ns | ns | ns | ns | * | ** | **** |
| **7** | **** | ** | ns | ns | ns | ns | - | ns | ns | ns | ns | ns | ** |
| **8** | **** | *** | * | ns | ns | ns | ns | - | ns | ns | ns | ns | * |
| **9** | **** | **** | ** | ns | ns | ns | ns | ns | - | ns | ns | ns | ns |
| **10** | **** | **** | *** | * | ns | ns | ns | ns | ns | - | ns | ns | ns |
| **11** | **** | **** | **** | *** | *** | * | ns | ns | ns | ns | - | ns | ns |
| **12** | **** | **** | **** | **** | *** | ** | ns | ns | ns | ns | ns | - | ns |
| **13** | **** | **** | **** | **** | **** | **** | ** | * | ns | ns | ns | ns | - |

1: Prompts to enter the presenting symptoms of the patient, 2: Prompts to enter the vital signs of the patient, 3: Ability to enter and/or automatically import results of laboratory tests ordered for the patient, 4: Ability to enter and/or automatically import results of medical images ordered for the patient, 5: Ability to enter other co-morbidities the patient might be suffering from, 6: Ability to enter all relevant information on prescription medications the patient is/was taking, 7: Ability to enter all relevant information on other non-prescription medications the patient is/was taking, 8: Ability to enter all relevant information on allergies to medications the patient suffered from, 9: Ability to enter all relevant information on adverse medication reactions the patient suffered from, 10: Ability to update patient's data and integrating new laboratory, imaging, and vital sign measurements, 11: Ability to transfer patient's data into the patient's electronic medical record, 12: Ability to enter information on congenital defects of the patient, 13: Ability to enter all relevant information on herbal medicines used by the patient

Comparisons were performed using ANOVA with Bonferroni's multiple comparisons, NS: not significant, *: *p*-value < 0.05, **: *p*-value < 0.01, ***: *p*-value < 0.001, ****: *p*-value < 0.0001.

**Additional Table S6:** Multiple comparisons of weights given by the panelists in the Analytic Hierarchy Process to each item in the quality of alerts category

| **Item #** | **1** | **2** | **3** | **4** | **5** | **6** | **7** | **8** | **9** | **10** | **11** | **12** |
| --- | --- | --- | --- | --- | --- | --- | --- | --- | --- | --- | --- | --- |
| **1** | - | ns | ** | **** | **** | **** | **** | **** | **** | **** | **** | **** |
| **2** | ns | - | ns | ns | **** | **** | **** | **** | **** | **** | **** | **** |
| **3** | ** | ns | - | ns | ** | **** | **** | **** | **** | **** | **** | **** |
| **4** | **** | ns | ns | - | ns | * | ** | **** | **** | **** | **** | **** |
| **5** | **** | **** | ** | ns | - | ns | ns | ns | * | ** | *** | **** |
| **6** | **** | **** | **** | * | ns | - | ns | ns | ns | ns | ns | * |
| **7** | **** | **** | **** | ** | ns | ns | - | ns | ns | ns | ns | ns |
| **8** | **** | **** | **** | **** | ns | ns | ns | - | ns | ns | ns | ns |
| **9** | **** | **** | **** | **** | * | ns | ns | ns | - | ns | ns | ns |
| **10** | **** | **** | **** | **** | ** | ns | ns | ns | ns | - | ns | ns |
| **11** | **** | **** | **** | **** | *** | ns | ns | ns | ns | ns | - | ns |
| **12** | **** | **** | **** | **** | **** | * | ns | ns | ns | ns | ns | - |

1: Suggestions and alerts should be evidence-based, provide a reference or references, and level of evidence, 2: Suggesting evidence-based and up-to-date recommendations, guidelines, and/or protocols to prescribe medications, 3: Alerts regarding allergy should distinguish between a serious potential allergy and minor side effect of the medication, 4: Alerts and suggestions should provide clear information on relative risk of harm for the given patient, 5: Ability to give warning when the prescribed dose differed from the recommended dose, 6: Ability to recommend evidence-based dosing frequency suitable for the patient, 7: Ability to recommend evidence-based duration of medication administration, 8: Clear instructions to guide prescribers on writing the reason for discontinuing or changing a medication order, 9: Prompts to indicate if additional charts other than the medication chart was used for the pediatric patient (for example other charts for intravenous fluids, nutrition, …etc.), 10: Clear instructions to obtain parent/guardian authorization to allow for immunization as per the national program, in case, immunization was due for a pediatric patient, 11: The system should not allow the use of non-standard abbreviations/nomenclature, 12: Compulsory review of medications prescribed before saving and validating orders

Comparisons were performed using ANOVA with Bonferroni's multiple comparisons, NS: not significant, *: *p*-value < 0.05, **: *p*-value < 0.01, ***: *p*-value < 0.001, ****: *p*-value < 0.0001.

**Additional Table S7:** Multiple comparisons of weights given by the panelists in the Analytic Hierarchy Process to each item in the admission and discharge of the patient category

| **Item #** | **1** | **2** | **3** | **4** | **5** | **6** | **7** | **8** | **9** | **10** | **11** |
| --- | --- | --- | --- | --- | --- | --- | --- | --- | --- | --- | --- |
| **1** | - | ns | * | ** | ** | **** | **** | **** | **** | **** | **** |
| **2** | ns | - | ns | ns | ns | * | ** | **** | **** | **** | **** |
| **3** | * | ns | - | ns | ns | ns | ns | ** | *** | **** | **** |
| **4** | ** | ns | ns | - | ns | ns | ns | * | ** | *** | **** |
| **5** | ** | ns | ns | ns | - | ns | ns | ns | ** | *** | *** |
| **6** | **** | * | ns | ns | ns | - | ns | ns | ns | * | ** |
| **7** | **** | ** | ns | ns | ns | ns | - | ns | ns | ns | * |
| **8** | **** | **** | ** | * | ns | ns | ns | - | ns | ns | ns |
| **9** | **** | **** | *** | ** | ** | ns | ns | ns | - | ns | ns |
| **10** | **** | **** | **** | *** | *** | * | ns | ns | ns | - | ns |
| **11** | **** | **** | **** | **** | *** | ** | * | ns | ns | ns | - |

1: The hospital's admission number assigned to the patient at the time of admission, 2: The date on which the patient was admitted to the hospital, 3: Name(s) of the ward(s) to which the patient was (were) admitted, 4: The name of the physician under whose care the patient was admitted to the hospital, 5: The date on which the patient was discharged from the hospital, 6: The name of the physician who decided to discharge the patient, 7: Bed(s) number(s) that was (were) assigned to the patient during their admission to the hospital, 8: Name of the hospital to which the patient was admitted, 9: Name of the physician who entered the patient information and verified that all details were correct, 10: The time on which the patient was discharged from the hospital, 11: The time on which the patient was admitted to the hospital

Comparisons were performed using ANOVA with Bonferroni's multiple comparisons, NS: not significant, *: *p*-value < 0.05, **: *p*-value < 0.01, ***: *p*-value < 0.001, ****: *p*-value < 0.0001.

**Additional Table S8:** Multiple comparisons of weights given by the panelists in the Analytic Hierarchy Process to each item in the general features category

| **Item #** | **1** | **2** | **3** | **4** | **5** | **6** | **7** | **8** | **9** |
| --- | --- | --- | --- | --- | --- | --- | --- | --- | --- |
| **1** | - | * | ** | **** | **** | **** | **** | **** | **** |
| **2** | * | - | ns | * | **** | **** | **** | **** | **** |
| **3** | ** | ns | - | ns | **** | **** | **** | **** | **** |
| **4** | **** | * | ns | - | ns | ** | **** | **** | **** |
| **5** | **** | **** | **** | ns | - | ns | ** | *** | **** |
| **6** | **** | **** | **** | ** | ns | - | ns | * | ** |
| **7** | **** | **** | **** | **** | ** | ns | - | ns | ns |
| **8** | **** | **** | **** | **** | *** | * | ns | - | ns |
| **9** | **** | **** | **** | **** | **** | ** | ns | ns | - |

1: The system should be as user friendly as practically possible providing easy to use interfaces, 2: Alerts should be clear and specify exactly why they were displayed, 3: The system should provide a prepackaged entry forms allowing accurate and comprehensive patient assessment, 4: The system should allow retrieval and viewing of all and/or selected patient's specific information as the user desires, 5: Users should provide reasons when opting to over-ride system recommendations, 6: Provided entries should be customizable in case the user needed to modify some of them, 7: Ability to remind the user to complete tasks and activities that were not completed or selected for follow up, 8: Users should be able to decline suggested recommendations, 9: Alerts and suggestions should pop-up when really necessary to avoid prescriber alert desensitization

Comparisons were performed using ANOVA with Bonferroni's multiple comparisons, NS: not significant, *: *p*-value < 0.05, **: *p*-value < 0.01, ***: *p*-value < 0.001, ****: *p*-value < 0.0001.

**Additional Table S9:** Multiple comparisons of weights given by the panelists in the Analytic Hierarchy Process to each item in the diseases and making diagnosis category

| **Item #** | **1** | **2** | **3** |
| --- | --- | --- | --- |
| **1** | - | * | *** |
| **2** | * | - | ns |
| **3** | *** | ns | - |

1: Ability to enter diagnosis, 2: Ability to access to offline, online, and searchable databases and references related to diseases and differential diagnosis, 3: Ability to provide hints for potential diagnosis based on the data entered into the assessment section

Comparisons were performed using ANOVA with Bonferroni's multiple comparisons, NS: not significant, *: *p*-value < 0.05, **: *p*-value < 0.01, ***: *p*-value < 0.001, ****: *p*-value < 0.0001.
